# HigB of *Pseudomonas aeruginosa* Enhances Killing of Phagocytes by Up-Regulating the Type III Secretion System in Ciprofloxacin Induced Persister Cells

**DOI:** 10.3389/fcimb.2016.00125

**Published:** 2016-10-14

**Authors:** Mei Li, Yuqing Long, Ying Liu, Yang Liu, Ronghao Chen, Jing Shi, Lu Zhang, Yongxin Jin, Liang Yang, Fang Bai, Shouguang Jin, Zhihui Cheng, Weihui Wu

**Affiliations:** ^1^State Key Laboratory of Medicinal Chemical Biology, Key Laboratory of Molecular Microbiology and Technology of the Ministry of Education, Department of Microbiology, College of Life Sciences, Nankai UniversityTianjin, China; ^2^Singapore Centre for Environmental Life Sciences Engineering, Nanyang Technological UniversitySingapore, Singapore; ^3^School of Biological Sciences, Division of Structural Biology and Biochemistry, Nanyang Technological UniversitySingapore, Singapore; ^4^State Key Laboratory of Medicinal Chemical Biology and College of Pharmacy and Life Sciences, Nankai UniversityTianjin, China; ^5^Department of Molecular Genetics and Microbiology, College of Medicine, University of FloridaGainesville, FL, USA

**Keywords:** toxin/antitoxin, type III secretion system, persistence, *Pseudomonas aeruginosa*, gene regulation

## Abstract

Bacterial persister cells are dormant and highly tolerant to lethal antibiotics, which are believed to be the major cause of recurring and chronic infections. Activation of toxins of bacterial toxin-antitoxin systems inhibits bacterial growth and plays an important role in persister formation. However, little is known about the overall gene expression profile upon toxin activation. More importantly, how the dormant bacterial persisters evade host immune clearance remains poorly understood. Here we demonstrate that a *Pseudomonas aeruginosa* toxin-antitoxin system HigB-HigA is required for the ciprofloxacin induced persister formation. Transcriptome analysis of a *higA*::Tn mutant revealed up regulation of type III secretion systems (T3SS) genes. Overexpression of HigB increased the expression of T3SS genes as well as bacterial cytotoxicity. We further demonstrate that wild type bacteria that survived ciprofloxacin treatment contain higher levels of T3SS proteins and display increased cytotoxicity to macrophage compared to vegetative bacterial cells. These results suggest that *P. aeruginosa* accumulates T3SS proteins during persister formation, which can protect the persister cells from host clearance by efficiently killing host immune cells.

## Introduction

Bacterial persisters are rare cells in a bacterial population that are tolerant to lethal antibiotics. Formation of persisters has been observed in almost all bacterial species investigated (Lewis, 2010). Persistence as a phenotypic switch is pre-existing in bacterial populations, with the characteristic of dormancy or slow growth. Reinoculation of the persister cells results in a similar heterogeneous population in which a subpopulation is tolerant to antibiotics. Formation of persister cells is influenced by environmental stresses and growth phases (Balaban et al., 2004; Keren et al., 2004; Dörr et al., 2009, 2010; Bernier et al., 2013; Helaine et al., 2014).

Toxin–antitoxin (TA) systems, composed of a toxin and a cognate antitoxin, play important roles in persister formation (Kim et al., 2011; Germain et al., 2013; Maisonneuve et al., 2013; Helaine et al., 2014; Verstraeten et al., 2015). Toxins can inhibit bacterial protein synthesis, DNA replication, cell wall synthesis or depolarize membrane, resulting in slow growth or dormant persister cells (Page and Peti, 2016). Toxins can be activated by various stimulations. For example, environmental stresses, such as starvation, induce the synthesis of bacterial alarmones guanosine tetraphosphate (ppGpp) and guanosine pentaphosphate (pppGpp), which trigger the degradation of antitoxins by proteases, resulting in activation of toxins (Nguyen et al., 2011; Maisonneuve et al., 2013). In addition, fluoroquinolones and oxidative stresses can activate toxins and induce persister formation through SOS response (Dörr et al., 2009, 2010; Wu et al., 2012).

Bacterial persisters are believed to be responsible for recurrent and chronic infections, due to the failure of antibiotics to eradicate the bacterial pathogens (Lewis, 2007). However, the mechanism by which the rare dormant persister cells evade the killing by host phagocytes remains poorly understood. Although it is believed that persister cells embedded in biofilm are shielded from host phagocytes (Leid, 2009), whether free persister cells are capable of surviving the attack of immune cells is not known. Numerous studies have revealed roles of TA systems in the regulation of bacterial gene expression, including virulence factors (Bertram and Schuster, 2014; Wen et al., 2014). Therefore, studies on the functions of TA systems will facilitate the understanding of the physiology of persister cells as well as their survival strategies within the host environments.

*Pseudomonas aeruginosa* is an opportunistic pathogen that causes acute and chronic infections in human (Balasubramanian et al., 2013). In *P. aeruginosa* strain PA14, two potential toxin-antitixoin systems have been identified, namely RelE-RelB and HigB-HigA (Williams et al., 2011; Wood and Wood, 2016). It has been demonstrated that HigB is a RNase, which cleaves mRNAs (Hurley and Woychik, 2009; Schureck et al., 2015, 2016a,b; Wood and Wood, 2016). In this study, we demonstrate that the toxin HigB contributes to persister formation. RNA-seq results revealed up regulation of type III secretion system (T3SS) genes in a *higA*::Tn mutant. The T3SS is a needle-like apparatus conserved in Gram negative pathogenic bacteria, through which effector proteins are directly translocated into the host cells, altering cell signaling or causing cell death. In *P. aeruginosa*, the T3SS plays an essential role in bacterial pathogenesis by killing phagocytes (Hauser, 2009; Diaz and Hauser, 2010). Consistent with the T3SS gene expression pattern, the *higA*::Tn mutant displayed higher cytotoxicity than the wild type strain. As expected, overexpression of the HigB resulted in a similar phenotype. These results imply a high level of cytotoxicity of the persister cells. Indeed, compared to vegetative cells, cells that survived short term ciprofloxacin treatment displayed increased cytotoxicity, which depends on HigB mediated up regulation of the T3SS. To our knowledge, this is the first demonstration of a connection between the HigB-HigA system and the T3SS. Our results suggest that T3SS proteins get accumulated during the process of persister formation, enabling the bacterial persisters to survive host clearance by actively killing the host immune cells.

## Results

### HigA negatively regulates the *higB-higA* operon

**Figure 1:**
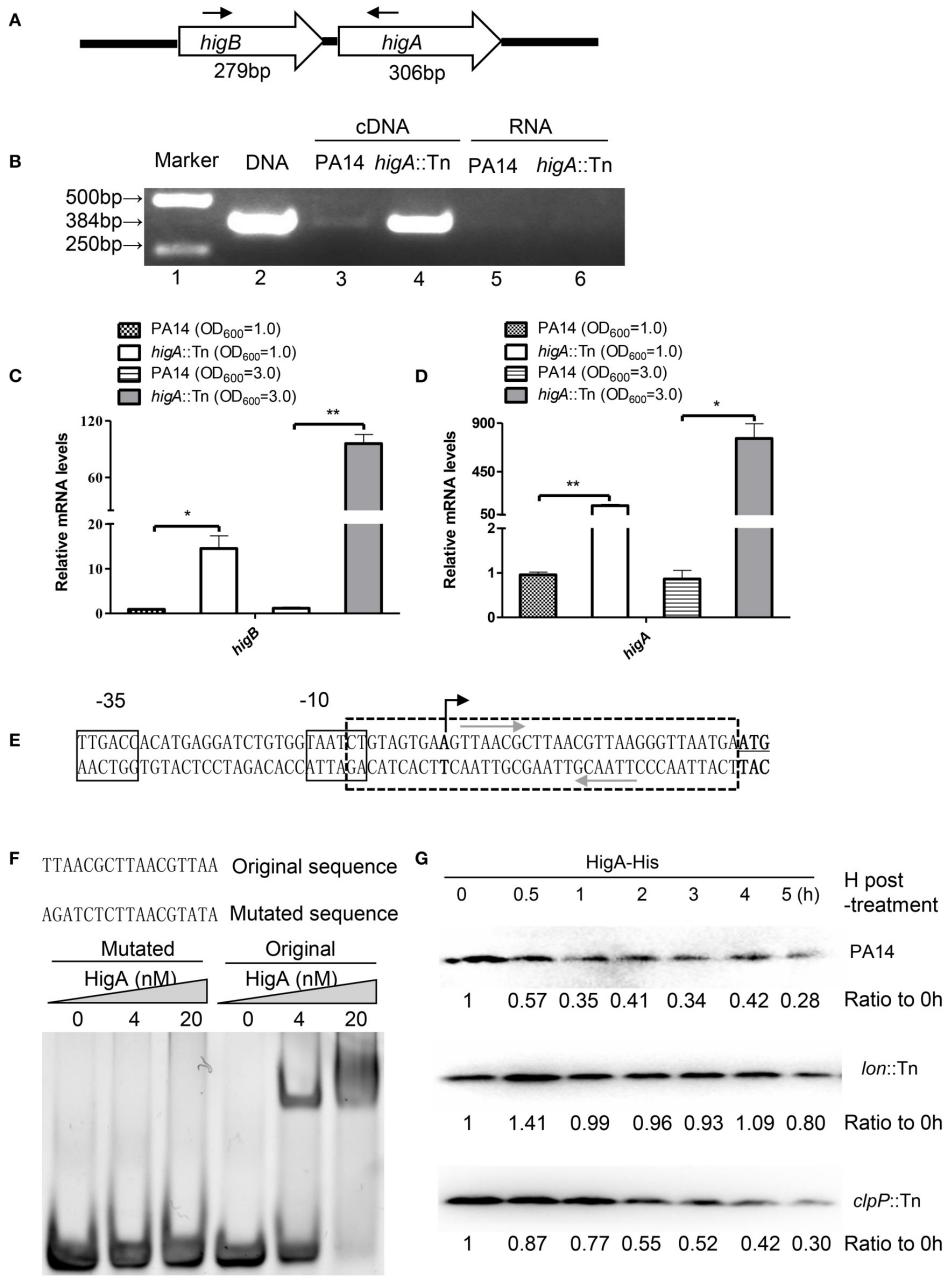
**HigA negatively regulates the *higB*-*higA* operon. (A)** Sketch map of the *higB*-*higA* operon. Arrows indicate the directions and locations of the primers for RT-PCR. **(B)** Total RNA was isolated from PA14 and the *higA*::Tn mutant. cDNA was synthesized and used as templates in PCR. RNAs were used in RT-PCR as negative controls. **(C,D)** The relative mRNA levels of *higB* and *higA* genes in PA14 and the *higA*::Tn mutant. Total RNA was isolated and the mRNA levels were determined by quantitative RT-PCR with the 16S rRNA as the internal control. Data represents the mean ± standard deviation from three independent experiments performed in triplicate. ^*^p < 0.05; ^**^p < 0.01 compared to wild type PA14 by Student's *t*-test. **(E)** Promoter region of the *higB*-*higA* operon. The predicted −10 and −35 elements of the promoter are boxed. The transcriptional start site is indicated by a black arrow, and the start codon of *higB* is underlined. The palindromic sequences of hypothetical HigA binding sites are indicated by gray arrows. **(F)** EMSA displaying binding of HigA to the *higB*-*higA* promoter. Purified HigA-His protein was incubated with the 38-bp DNA fragment indicated by the box with dashed lines in **(E)** or altered sequence. The mixtures were electrophoresed and observed by ethidium bromide staining. **(G)** Cleavage of HigA by the Lon protease. Wild type PA14, the *clpP*::Tn and *lon*::Tn mutants carrying pMMB67EH-*higA*-His were cultured in the presence of 1 mM IPTG for 1 h. Then 50 μg/ml spectinomycin was added to the medium. At indicated time points, the HigA-His levels were determined by Western blot analysis with an anti-His antibody. The relative density of each band was determined with Image J.

A recent study identified the open reading frame of HigB in PA14 and demonstrated its growth inhibitory function (Wood and Wood, 2016). In most type II TA systems, toxin and antitoxin genes form one operon and the antitoxin binds to and represses its own promoter (Wood and Wood, 2016). To test whether *higB* and *higA* are in the same operon, we designed a pair of primers annealing to the 5′ end of *higB* and 3′ end of *higA* coding region, respectively (Figure 1A), and performed RT-PCR. Total RNA was isolated from PA14 and a *higA* mutant from the PA14 transponson insertion mutant library (Liberati et al., 2006). A 384-bp PCR product was amplified using cDNA from the *higA*::Tn mutant (Figure 1B, lane 4), and the size was the same as that when genomic DNA was used as the template (Figure 1B, lane 2). Substantially less PCR product was obtained when cDNA from wild type PA14 was used as the template (Figure 1B, lane 3), suggesting a lower HigB mRNA level. To confirm the transcriptional level of *higB* and *higA*, we performed quantitative RT PCR with previously reported PA1769 and *proC* as internal controls for normalization (Savli et al., 2003; Son et al., 2007). Since HigB might cleave mRNAs and affect the expression of multiple genes, we included the 16S rRNA (PA0668.1) (Ruzin et al., 2007), which might not be a target of HigB, as another internal control. Similar mRNA fold of changes (within 1.5-fold difference) were observed when *proC* and the 16S rRNA were used as internal controls. Therefore, we used the 16S rRNA as the internal control in this study. At both exponential and stationary growth phases, the mRNA levels of *higB* and *higA* in the *higA*::Tn mutant were higher than those in wild type PA14 (Figures 1C,D). In addition, a previous microarray analysis has demonstrated an up regulation of *higB* in a *higA* mutant (Wood and Wood, 2016). In combination, these results suggest that *higB* and *higA* are in the same operon, which is negatively regulated by the HigA.

To examine whether HigA binds to the promoter of its own operon, we first determined the transcriptional start site by a 5′ RACE analysis. The start site was located at 29 bp upstream of the start codon for *higB* (Figure 1E). Of note, we found a palindromic sequence downstream of the −10 region (Figure 1E), which might be the binding site of HigA. Electrophoretic mobility shift assay (EMSA) revealed an interaction between the fragment and HigA, and mutation of the palindromic sequence abolished the interaction (Figure 1F). These results suggest that HigA directly binds to and represses the promoter of the *higB*-*higA* operon.

### The Lon protease contributes to the degradation of HigA

To identify which protease is involved in the degradation of HigA, a C-terminus His-tagged HigA (HigA-His) driven by a *tac* promoter was introduced into wild type PA14. After 60 min of culture in the presence of IPTG, spectinomycin was added to block protein synthesis, then the stability of HigA-His was monitored. In wild type PA14 and a *clpP*::Tn mutant, the HigA-His was gradually degraded. However, the protein was stable in a *lon*::Tn mutant (Figure 1G, Figure S1A), suggesting a role of the Lon protease in the degradation of HigA.

### HigB-HigA regulates persister formation in PA14

**Figure 2:**
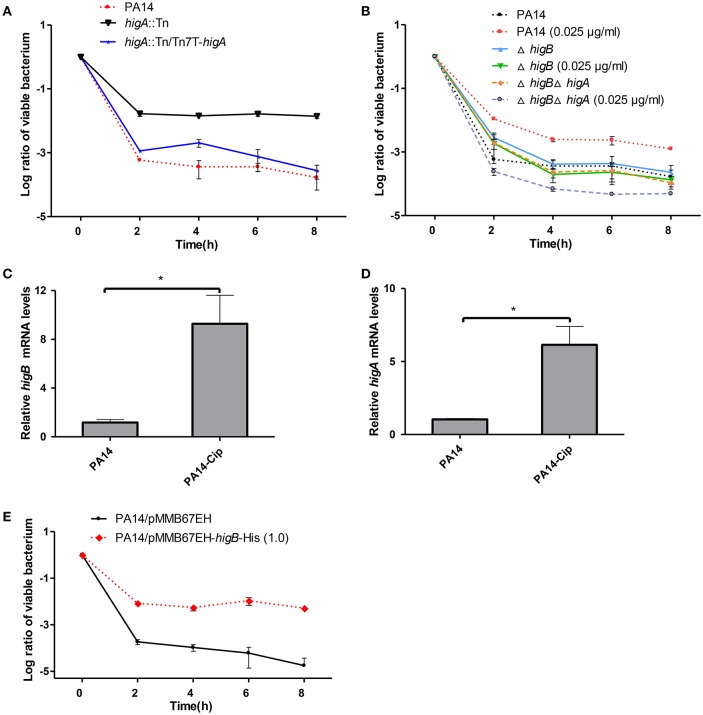
**Role of HigA-HigB in persister formation. (A)** Wild type PA14, a *higA*::Tn mutant and a complemented strain were treated with 0.25 μg/ml ciprofloxacin. At indicated time points the survival rates were determined by plating assay. **(B**) Wild type PA14, the Δ*higA* and Δ*higA*Δ*higB* mutants were cultured in the presence or absence of 0.025 μg/ml ciprofloxacin for 2 h and then treated with 0.25 μg/ml ciprofloxacin. The survival rates were determined by plating assay. **(C,D)** Wild type PA14 was treated with 0.025 μg/ml ciprofloxacin for 2 h and the mRNA levels of *higB* or *higA* were determined by quantitative RT-PCR. Error bars represent the standard error. ^*^p < 0.05, by Student's *t*-test. **(E)** Wild type PA14 carrying a P_*tac*_ driven *higB* gene or the empty vector were cultured with 1 mM IPTG for 2 h, followed by treatment with 0.25 μg/ml ciprofloxacin. The survival rates were determined by plating. Error bars represent the standard errors. The graphs are representatives of three independent experiments.

To test the role of HigB-HigA in persister formation, the *higA*::Tn mutant was examined for a time-dependent killing by ciprofloxacin. Compared to the wild type PA14, the *higA*::Tn mutant displayed 100-fold higher survival rate, which was restored to the wild type level by complementation with an intact *higA* gene (Figure 2A). However, a Δ*higB* mutant displayed a similar survival rate as the wild type PA14 (Figure 2B), which we suspect might be due to redundant TA systems in *P. aeruginosa*. It has been demonstrated that sublethal level of ciprofloxacin induces persister formation (Dörr et al., 2009, 2010). Thus, we examined the role of HigB in ciprofloxacin induced persister formation as previously described (Dörr et al., 2009, 2010). Pre-exposure to 0.025 μg/ml (1/10 MIC) ciprofloxacin increased the survival rate of wild type PA14 by approximately 5-fold, suggesting an induction of persister formation (Figure 2B). However, deletion of *higB* or the *higB*-*higA* operon abolished such induction (Figure 2B). The expression of *higB* and *higA* was induced by the ciprofloxacin treatment (Figures 2C,D) and overexpression of HigB increased bacterial survival rate by approximately 1000-fold (Figure 2E). In addition, the minimal inhibitory concentration (MIC) of ciprofloxacin was not altered by the mutation of *higA* or overexpression of *higB* (data not shown). In combination, these results demonstrate that HigB contributes to persister formation.

### Transcriptome analysis of the *higA*::Tn mutant

RNA-seq analyses were employed to explore the effect of *higA* inactivation on bacterial global gene expression at both exponential and stationary growth phases. Compared to wild type PA14, expression of 193 genes was altered in the *higA* mutant at both growth phases (Table S1). Of note, all of the T3SS genes were up regulated in the *higA* mutant (Table 1), suggesting a regulatory role of the TA system on the T3SS.

**Table 1:** **mRNA levels of T3SS genes in the *higA*::Tn mutant compared to those in wild type PA14**.

**Locus Tag PA14**	**Locus Tag PAO1**	**Name**	**Product**	**Fold changes *higA*::Tn/WT (E)**	***P*-value**	**Fold changes *higA*::Tn/WT (S)**	***P*-value**
PA14_RS17315	PA1690	*pscU*	Translocation protein in type III secretion	5.040	5.03E-18	3.733	1.51E-12
PA14_RS17310	PA1691	*pscT*	Translocation protein in type III secretion	6.569	5.63E-16	3.901	2.57E-08
PA14_RS17305	PA1692		Probable translocation protein in type III secretion	4.022	3.45E-05	5.280	4.73E-06
PA14_RS17300	PA1693	*pscR*	Translocation protein in type III secretion	4.259	2.16E-13	4.456	6.79E-11
PA14_RS17295	PA1694	*pscQ*	Translocation protein in type III secretion	4.940	9.64E-21	5.341	1.30E-21
PA14_RS17285	PA1696	*pscO*	Translocation protein in type III secretion	6.773	4.84E-13	7.603	1.83E-16
PA14_RS17275	PA1698	*popN*	Type III secretion outer membrane protein PopN precursor	9.668	1.99E-17	10.303	2.25E-19
PA14_RS17270	PA1699		Pcr1	7.646	6.87E-22	10.891	3.05E-29
PA14_RS17265	PA1700		Pcr2	9.520	3.65E-12	8.711	1.06E-11
PA14_RS17260	PA1701		Pcr3	15.069	3.35E-26	4.898	6.08E-11
PA14_RS17255	PA1702		Pcr4	7.637	1.40E-07	3.461	5.32E-03
PA14_RS17250	PA1703	*pcrD*	Type III secretory apparatus protein PcrD	8.296	2.55E-36	4.419	8.01E-21
PA14_RS17245	PA1704	*pcrR*	Transcriptional regulator protein PcrR	10.180	1.10E-07	3.673	1.04E-03
PA14_RS17190	PA1715	*pscB*	Type III export apparatus protein	10.823	2.91E-27	7.750	1.25E-22
PA14_RS17185	PA1716	*pscC*	Type III secretion outer membrane protein PscC precursor	7.545	8.13E-34	5.668	5.06E-26
PA14_RS17180	PA1717	*pscD*	Type III export protein PscD	8.176	8.09E-17	6.118	5.71E-14
PA14_RS17175	PA1718	*pscE*	Type III export protein PscE	3.671	3.44E-10	5.666	1.50E-18
PA14_RS17170	PA1719	*pscF*	Type III export protein PscF	3.486	4.18E-07	6.825	4.20E-14
PA14_RS17165	PA1720	*pscG*	Type III export protein PscG	4.787	3.27E-17	6.694	4.94E-24
PA14_RS17160	PA1721	*pscH*	Type III export protein PscH	5.776	2.08E-19	5.984	2.64E-19
PA14_RS17155	PA1722	*pscI*	Type III export protein PscI	5.717	5.44E-22	4.898	2.78E-19
PA14_RS17150	PA1723	*pscJ*	Type III export protein PscJ	6.078	7.63E-27	5.100	1.98E-22
PA14_RS17145	PA1724	*pscK*	Type III export protein PscK	10.686	3.18E-32	5.262	2.00E-14
PA14_RS17140	PA1725	*pscL*	Type III export protein PscL	7.367	4.46E-30	5.816	2.25E-22
PA14_RS17240	PA1705	*pcrG*	Regulator in type III secretion	10.210	4.38E-09	7.620	4.03E-08
PA14_RS17235	PA1706	*pcrV*	Type III secretion protein PcrV	6.118	4.35E-30	9.030	5.90E-44
PA14_RS17230	PA1707	*pcrH*	Regulatory protein PcrH	6.341	9.18E-29	19.662	1.89E-73
PA14_RS17225	PA1708	*popB*	Translocator protein PopB	5.914	1.39E-21	10.593	1.10E-37
PA14_RS17220	PA1709	*popD*	Translocator outer membrane protein PopD precursor	4.516	3.14E-12	6.590	5.54E-20
PA14_RS17215	PA1710	*exsC*	ExsC, exoenzyme S synthesis protein C precursor.	4.807	1.56E-23	6.655	3.21E-34
PA14_RS17210	PA1711		ExsE	4.700	2.32E-05	5.277	1.48E-06
PA14_RS17205	PA1712	*exsB*	Exoenzyme S synthesis protein B	5.985	1.56E-21	5.144	5.97E-22
PA14_RS17200	PA1713	*exsA*	Transcriptional regulator ExsA	5.374	4.24E-24	4.824	4.09E-22
PA14_RS17195	PA1714		ExsD	6.886	2.26E-16	9.658	9.68E-24
PA14_RS00230	PA0044	*exoT*	Exoenzyme T	6.141	4.01E-28	8.190	3.39E-41
PA14_RS14785	PA2191	*exoY*	Adenylate cyclase ExoY	6.589	1.35E-30	6.053	1.75E-31
PA14_RS20960		*exoU*	ExoU	4.443	1.56E-21	8.603	1.97E-44
PA14_RS20955		*spcU*	SpcU	3.093	9.69E-10	4.960	1.31E-20
PA14_RS05730	PA3842	*spcS*	SpcS	6.434	1.11E-08	11.384	4.76E-15

*E, exponential growth phase*.

*S, stationary growth phase*.

### Increased expression of T3SS genes and cytotoxicity of the *higA*::Tn mutant

**Figure 3:**
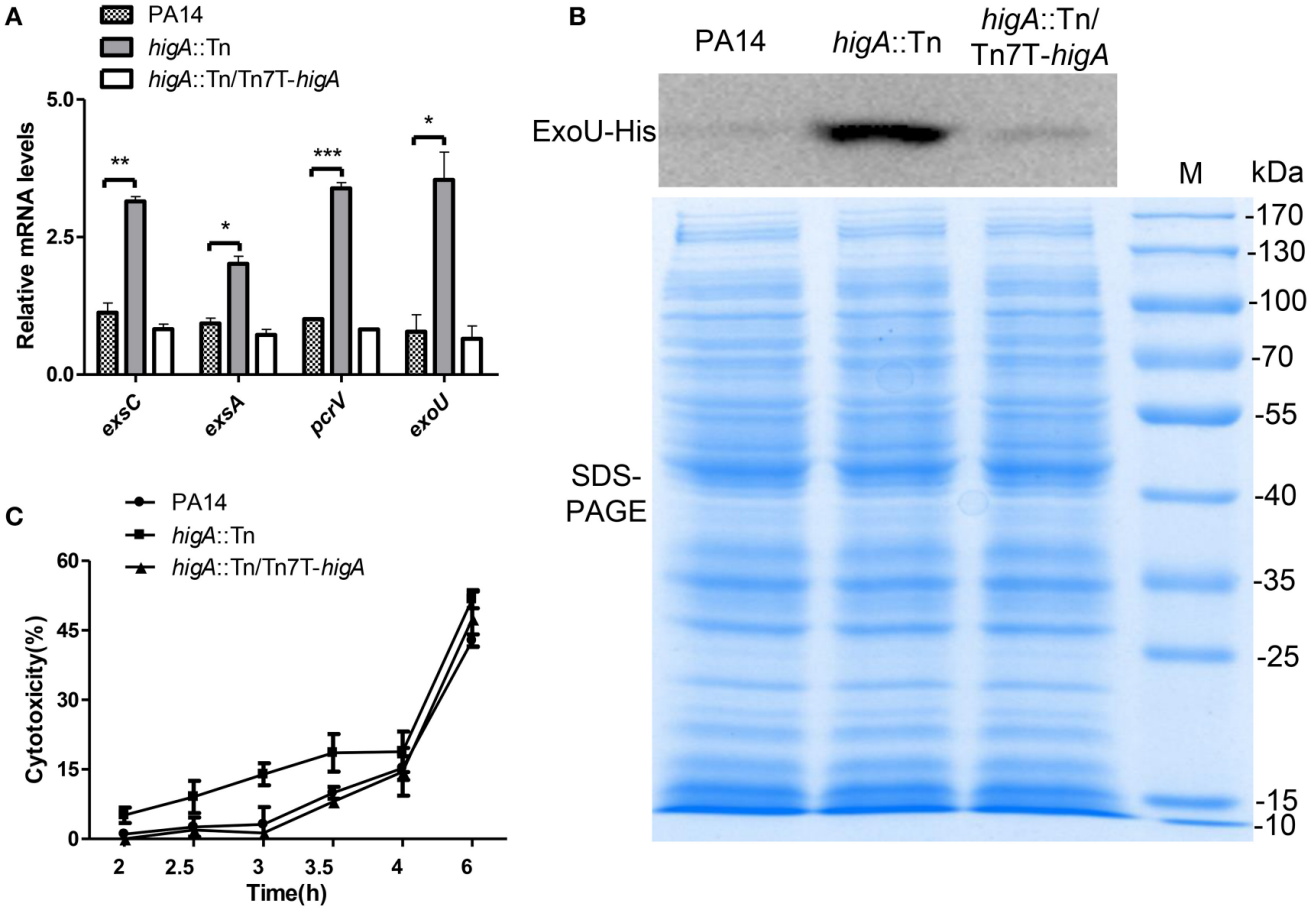
**Expression levels of T3SS genes in wild type PA14, the *higA*::Tn mutant and complemented strain. (A)** Relative mRNA levels of *exsC, exsA, pcrV*, and *exoU* in indicated strains at exponential growth phase (OD_600_ = 0.8~1.0). Error bars represent the standard errors. ^*^p < 0.05, ^**^p < 0.01, ^***^p < 0.005 compared to wild type PA14 by Student's *t*-test. **(B)** Bacteria carrying an *exoU*-His driven by its native promoter (P_*exoU*_*-exoU*-His) were grown in LB at 37°C. At the OD_600_ of 1.0, bacteria were collected. Samples from equivalent bacterial cells were loaded into SDS-PAGE gels and stained with Coomassie blue or probed with an anti-His antibody. **(C)** Raw264.7 cell were infected by indicated strains at an MOI of 10. At indicated time points, the relative cytotoxicity was determined by the LDH release assay.

To confirm the elevated expression of T3SS genes in the *higA*::Tn mutant, the mRNA levels of *exsA, exsC* (two positive regulatory genes), *pcrV* (required for translocation of effector proteins) and *exoU* (encodes for an effector protein) were examined. Mutation of *higA* resulted in higher mRNA levels of all of these genes, which were restored to the wild type levels by complementation with a *higA* gene (Figure 3A, Figure S1B). As the *higA*::Tn mutant grew slower than the wild type strain, translation of the T3SS genes might be impeded. To test this possibility, we examined PcrV protein levels by immunostaining in strains harboring a *mcherry* gene driven by the promoter of *higB-higA* (P_*higB*_*-mcherry*). Compared to the wild type strain, the *higA*::Tn mutant expressed higher levels of PcrV and mCherry proteins (Figure S1C). Next, we constructed a C-terminal His-tagged ExoU driven by its native promoter (P_*exoU*_-ExoU-His). Consistent with the above results, the levels of ExoU-His protein in the *higA*::Tn mutant were higher at both exponential (Figure 3B) and stationary growth phases (Figure S1D).

To test whether the increased expression of T3SS genes leads to higher cytotoxicity, we performed LDH release assay. Compared to wild type PA14, the *higA*::Tn mutant caused quicker cell death to either macrophages (Raw264.7) (Figure 3C) or epithelial cells (HeLa) (Figure S1E). In addition, when HeLa cells were infected with strains containing the ExoU-His, more ExoU was translocated into the cells by the *higA*::Tn mutant (Figures S1F,G). Altogether, these results demonstrate that mutation of the *higA* results in up regulation of T3SS genes and consequently higher cytotoxicity.

### Activation of HigB increases the expression of T3SS genes and cytotoxicity

**Figure 4:**
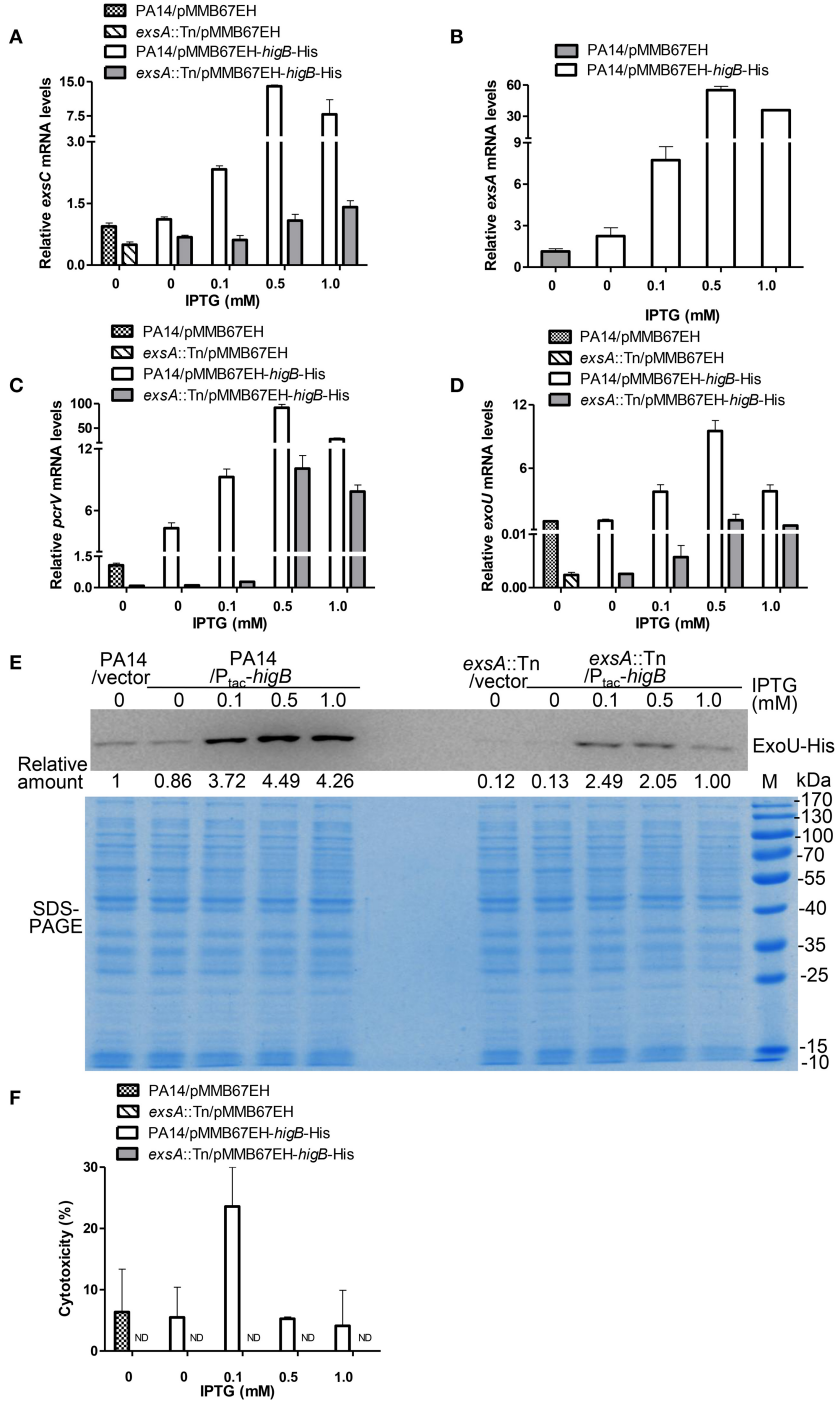
**HigB promotes T3SS mediated cytotoxicity**. Wild type PA14 and an *exsA*::Tn mutant containing pMMB67EH-*higB*-His or pMMB67EH was grown in the presence of indicated concentrations of IPTG for 3 h. Quantitative RT-PCR was used to determine the relative mRNA levels of *exsC* **(A)**, *exsA* **(B)**, *pcrV* **(C)**, and *exoU* **(D)**. **(E)** PA14 or *exsA*::Tn containing *exoU*-His driven by its native promoter (P_*exoU*_*-exoU*-His) and pMMB67EH-*higB* or pMMB67EH was grown in the presence of indicated concentrations of IPTG for 3 h. Samples from equivalent bacterial cells were loaded into SDS-PAGE gels and stained with Coomassie blue or or probed with an anti-His antibody. The relative density of each band was determined by Image J. **(F)** Raw264.7 cells were infected with the bacteria at an MOI of 10 for 3.5 h, followed by LDH release assay. ND, not detectable.

HigB functions as a RNase, which is directly inhibited by HigA (Wood and Wood, 2016). Thus, we examined the role of HigB in the expression of T3SS genes and cytotoxicity. First, a Δ*higB*Δ*higA* double mutant was constructed, which displayed similar levels of T3SS gene expression and cytotoxicity as the wild type PA14 (Figures S2A,B). Second, a C-terminal His-tagged HigB driven by a regulatable *tac* promoter (P*tac*-*higB*-His) or the empty vector was introduced into wild type PA14 (Figure S2C). Addition of IPTG increased the mRNA levels of *exsC, exsA, pcrV*, and *exoU*, with the highest levels in the presence of 0.5 mM IPTG. In the presence of 1.0 mM IPTG, the mRNA levels of those genes were reduced, which might be due to strong growth inhibition as a consequence of high level expression of the HigB (Figures 4A–D). To further confirm the expression level of ExoU, we transferred the plasmid carrying P_*exoU*_-ExoU-His into the above strains. Consistently, the protein level of ExoU was increased by the overexpression of HigB (Figure 4E).

Next, we determined the correlation between bacterial cytotoxicity and expression levels of HigB. Bacteria grown in the presence 0.1 mM IPTG displayed the highest cytotoxicity to both Raw264.7 (Figure 4F) and HeLa cells (Figure S2D). However, further increase of the IPTG concentration reduced the cytotoxicity (Figure 4F, Figure S2D), although the mRNA levels of the T3SS genes were higher than or similar to those in the presence 0.1 mM IPTG (Figures 4A–D). Mutation of *exsA* severely reduced the HigB mediated increase of the T3SS gene expression and cytotoxicity (Figures 4A–F, Figure S2D). These results suggest that HigB promotes bacterial cytotoxicity through the T3SS. However, too high level of HigB might repress the overall bacterial fitness, which impedes the translocation of T3SS effector proteins.

### Cytotoxicity of persister cells

Our results from the *higA*::Tn mutant and the HigB overexpressing strain demonstrate that activation of HigB increases persister formation and T3SS mediated cytotoxicity. A more important question is whether persister cells harbor higher levels of T3SS proteins and are more cytotoxic than their isogenic vegetative cells.

In the bacterial survival assay, we noticed lysis of bacterial cells during ciprofloxacin treatment, presumably due to production and release of pyocins (Penterman et al., 2014; Sun et al., 2014). Based on this phenotype, we developed a method to collect persister cell by washing the ciprofloxacin treated bacteria with 0.3M sucrose, which could efficiently remove lysed cell debris. To assess the effectiveness of this method, we treated a wild type PA14 strain containing a *gfp* gene driven by the *higB* promoter (P_*higB*_-*gfp*) with 0.025 μg/ml ciprofloxin for 2 h to induce persister formation. Then the cells were incubated with 0.25 μg/ml ciprofloxacin for 6 h, resulting in a survival rate of 0.01% as determined by plating assay. Such treated bacterial cells were harvested by centrifugation and washed twice with 0.3M sucrose, followed by propidium iodide (PI) staining. As presented in Figure S3A, 93 ± 0.5% collected cells were PI negative, suggesting an effective isolation of persister cells. In addition, bacteria with strong green fluorescence were negative for PI staining, or vice versa (Figure S3A), indicating an up regulation of HigB in the persister cells. To examine the levels of PcrV in the persister cells, the collected bacterial cells were subjected to immunostaining with an anti-PcrV antibody. 79.7 ± 3.7% GFP positive cells were positive for PcrV (Figure S3B). In combination, these results demonstrate elevated levels of HigB and PcrV in persister cells.

**Figure 5:**
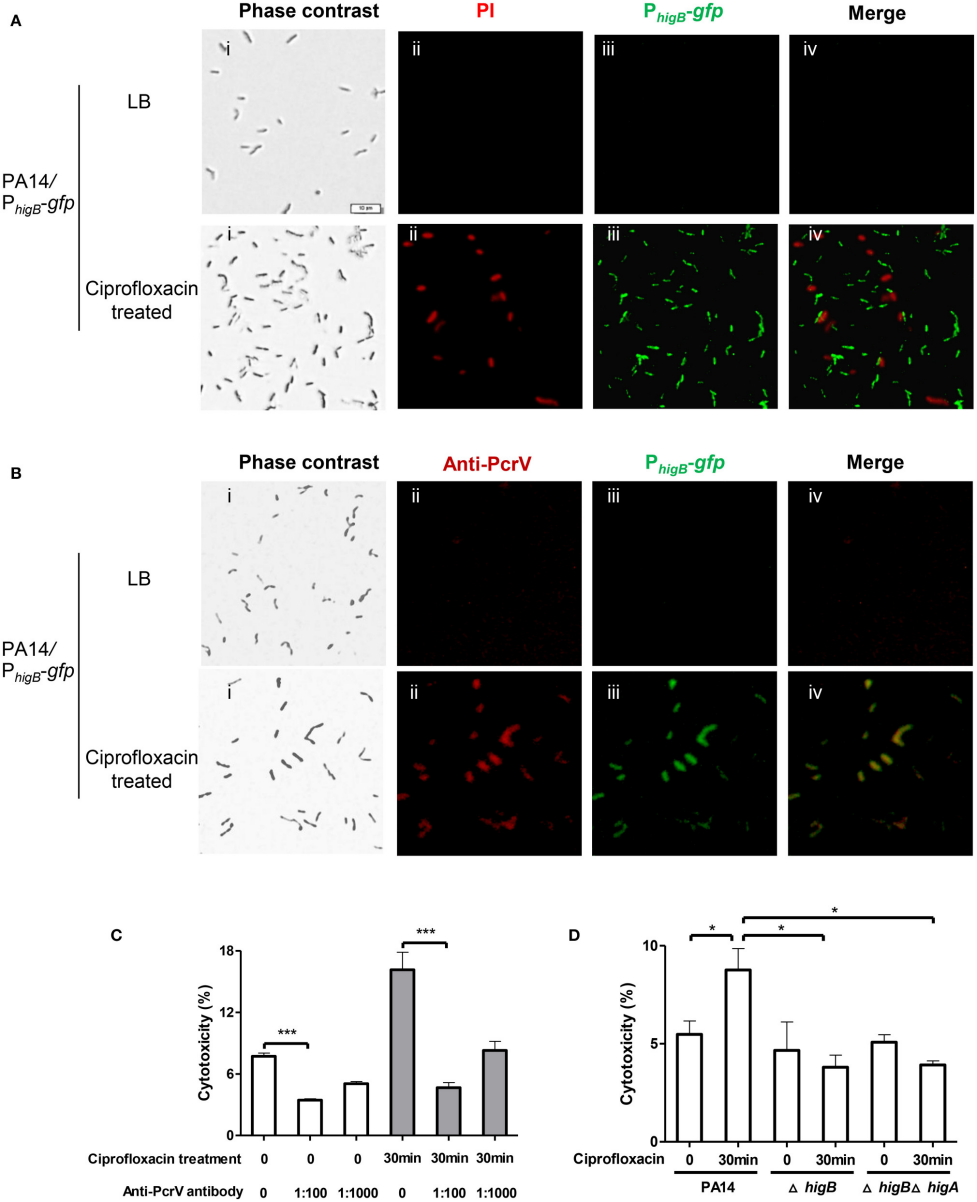
**Levels of HigB, PcrV and cytotoxicity of bacterial cells that survived ciprofloxacin treatment**. PA14/P_*higB*_-*gfp* was cultured in the presence of 0.025 μg/ml ciprofloxacin for 2 h and then treated with 0.125 μg/ml ciprofloxacin for 30 min. The bacteria were washed with PBS twice, stained with PI **(A)** or fixed and permeabilized and then stained with rabbit anti-PcrV, followed by Alex Fluor 594–labeled goat anti–rabbit immunoglobulin. Bar = 10 μm **(B)**. Quantification of fluorescence positive cells was based on analysis of about 100 cells from three different samples. **(C)** Wild type PA14 was cultured in the presence of 0.025 μg/ml ciprofloxacin for 2 h and then treated with 0.125 μg/ml ciprofloxacin for 30 min. Raw264.7 cells were infected with the surviving bacteria or bacteria grown in LB at an MOI of 10 for 3.5 h. The anti-PcrV antibody was added to the medium at indicated dilutions. The relative cytotoxicity was determined by the LDH release assay. Error bars represent the standard errors. **(D)** Wild type PA14, the Δ*higA* or Δ*higA*Δ*higB* mutant was cultured in the presence of 0.025 μg/ml ciprofloxacin for 2 h and then treated with 0.125 μg/ml ciprofloxacin for 30 min. Raw264.7 cells were infected with the survived bacteria or bacteria grown in LB at an MOI of 10 for 3.5 h. The relative cytotoxicity was determined by the LDH release assay. Error bars represent the standard errors. Each graph represents the results of three independent experiments. ^*^p < 0.05, ^***^p < 0.005 by Student's *t*-test.

Next, we examined the cytotoxicity of persister cells. Persister cells of wild type PA14 were collected as aforementioned and used to infect Raw264.7 cells. However, the persister cells displayed minimal cytotoxicity compared to vegetative cells (Figures S3C,D). We suspected that the 6-h exposure to ciprofloxacin might result in highly dormant cells that are unable to inject T3SS effectors. Therefore, we reduced the treatment time to 30 min, which resulted in 25% bacterial survival rate. As represented in Figure 5A, 83 ± 2.8% cells collected after ciprofloxacin treatment were PI negative. 89 ± 6.6% cells were GFP positive but GFP and PI double positive cell was barely observed (Figure 5A, lower panels), suggesting high levels of HigB in survived cells. Among the cells, 77 ± 10.1% were double positive for GFP and PcrV (Figure 5B, lower panels), whereas bacteria grown in LB were negative for GFP or PcrV (Figure 5B, upper panels). These results suggest that the expression levels of HigB and PcrV were higher in the survived bacterial cells than those in vegetative cells. These surviving bacteria displayed higher cytotoxicity to Raw264.7 cells (Figure 5C). Addition of anti-PcrV antibody, which has been demonstrated to protect cells from T3SS mediated cytotoxicity (Warrener et al., 2014), protected the Raw264.7 cells from killing by the bacteria survived of the ciprofloxacin treatment (Figure 5C).

To exclude the possibility that the macrophages were killed from stimulation by large amount of LPS or other bacterial ligands in the collected bacterial samples, we tested an *exsA*::Tn mutant strain. After the same ciprofloxacin treatment, the survival rate of the mutant was similar to that of wild type PA14, however, the bacteria displayed minimum cytotoxicity (Figures S3C,D). Next, we incubated wild type bacteria at 50°C for 30 min, which also resulted in 25% survival rate. The surviving bacteria barely caused cell death (Figures S3C,D). In combination, the above results demonstrate that compared to vegetative cells, bacteria that survived the 30 min ciprofloxacin treatment contain higher level of T3SS proteins, which leads to increased cytotoxicity.

### HigB contributes to the increased T3SS gene expression and cytotoxicity of bacteria survived ciprofloxacin treatment

To examine the role of HigB in the expression of T3SS genes in bacteria survived ciprofloxacin treatment, we treated either the Δ*higB* mutant or the Δ*higB*Δ*higA* mutant with 0.025 μg/ml ciprofloxin for 2 h followed by incubation with 0.125 μg/ml ciprofloxacin for 30 min. The expression levels of HigB and PcrV were examined by fluorescence microscopy as described above. Similar to wild type PA14, the promoter activity of *higB* was increased in the surviving bacteria (Figures S4, S5). The stronger fluoresce in the Δ*higB*Δ*higA* mutant further confirmed the negative regulatory role of the HigA on the *higB*-*higA* operon (Figures S4, S5). The levels of PcrV were significantly lower in the Δ*higB* or Δ*higB*Δ*higA* mutant than that in the wild type PA14 (Figures S4, S5). Consistently, the Δ*higB* or Δ*higB*Δ*higA* mutant cells survived ciprofloxacin treatment displayed lower cytotoxicity compared to the counterpart of wild type cells (Figure 5D). Therefore, HigB plays an important role in the up regulation of T3SS genes and increased cytotoxicity in survived bacteria.

## Discussion

In this study, we demonstrated that HigB is involved in ciprofloxacin induced persister formation and up regulation of the T3SS genes in *P. aeruginosa*. Mutation of *higA* or overexpression of *higB* did not alter the MIC of ciprofloxacin to the bacteria. Our RNA-seq results demonstrated no significant change in the expression levels of the multidrug efflux pumps in the *higA* mutant. Quantitative RT PCR results confirmed that the expression level of the major multidrug efflux pump MexAB-OprM was not altered in the *higA* mutant or the *higB* overexpression strain (data not shown) (Dreier and Ruggerone, 2015). However, the bacterial survival rate was significantly increased by the mutation of *higA* or overexpression of *higB* after ciprofloxacin treatment (Figures 2A,E), suggesting a role of HigA-HigB in persister formation.

Through a microarray analysis, Wood et al. found that mutation of *higA* reduced the expression of pyochelin biosynthesis genes (Wood and Wood, 2016). Our RNA-seq analysis of the *higA*::Tn mutant revealed similar expression pattern of those genes (Table S1). In addition, the whole T3SS gene clusters were up regulated, which we demonstrate to be dependent on HigB. The *P. aeruginosa* In *M. tuberculosis*, overexpression of HigB reduced the levels of a subset of mRNAs and increased HigB is the cleavage of tmRNA, which is involved in the rescue of ribosomes stalled on mRNAs (Christensen and Gerdes, 2003; Schuessler et al., 2013). It has been demonstrated in *E. coli* and *M. tuberculosis* that HigB associates with ribosome and cleaves mRNA at A-rich sequences (Hurley and Woychik, 2009; Schureck et al., 2015, 2016a,b). In addition, mutation of the *higA* gene did not lead to bacterial death (Wood and Wood, 2016 and our study). These results indicate that HigB might have a specific range of target mRNAs. As many genes contain A-rich codons, it is difficult to judge the target mRNAs solely based on the sequence. One of the possibilities is that the recognition of target mRNA or subsequent cutting by the HigB is affected by the movement of ribosome, i.e., the longer the ribosome stall at the A-rich codons, the more likely the mRNA is cleaved by HigB. As it has been demonstrated that ribosome stalling is affected by the amino acid sequence as well as environmental stimulations (Jin et al., 2016; Wilson et al., 2016), it will be interesting to examine the HigB mediated cleavage of the A-rich codons (such as AAA) with different neighboring sequences or under different conditions.

In this study, we used various concentrations of IPTG to induce ectopic expression of HigB in wild type PA14. With increasing expression of the HigB, the levels of T3SS gene expression and bacterial cytotoxicity rose and then dropped. Consistent with these observations, wild type PA14 that survived 0.5-h ciprofloxacin treatment displayed higher T3SS mediated cytotoxicity. However, bacteria that survived 6-h ciprofloxacin treatment displayed minimal cytotoxicity, although the PcrV level was higher than that in untreated bacteria. We hypothesize that the HigB recognizing sites might be overrepresented in the mRNA of a T3SS negative regulator, rendering it more vulnerable to HigB mediated cleavage. Of note, overexpression of HigB increased the expression levels of T3SS genes in an *exsA*::Tn mutant (Figures 4A–F). These results suggest that the HigB targeted T3SS regulator might repress the expression of T3SS genes independent of ExsA.

On the other hand, with higher levels of HigB, mRNAs with less HigB recognizing sites are also cleaved, thus reducing the overall biological fitness and the bacterial ability to respond to host cell contact. In addition, the assembly of T3SS apparatus or translocation of T3SS effectors might be impeded, thus leading to reduced cytotoxicity. Given the complicated environment in the host, each bacterium might encounter different levels of antibiotics. It might be possible that during persister formation, the levels of activated HigB are heterologous among the bacterial population. Moderate activation of HigB increases the expression of T3SS and bacterial cytotoxicity, while further up regulation and activation of HigB render the bacteria dormant and highly tolerant to antibiotics. It has been demonstrated in an animal model that T3SS-negative bacteria are protected from host clearance by the isogenic wild type strain, which actively kills phagocytes through the T3SS (Hauser, 2009; Diaz and Hauser, 2010; Czechowska et al., 2014). Therefore, we suspect that in the bacterial population that survived antibiotic treatment, bacteria with higher cytotoxicity might protect highly dormant cells from host immune cells, thus enable the survival of the persister cells. In biofilm, HigB in a small portion of bacteria might be activated, leading to persister formation as well as up regulation of T3SS genes. We previously found that the biomass of *P. aeruginosa* biofilm was reduced by ciprofloxacin treatment, suggesting dispersal of the biofilm (Sun et al., 2014). Therefore, it might be possible that bacteria inside biofilm are getting exposed to phagocytes during antibiotic treatment. In this scenario, the highly dormant persister cells might be protected by the T3SS proficient cells. It will be interesting to observe the expression levels of HigB and T3SS genes in individual cells inside biofilm with or without antibiotic treatment.

Recently, Pu et al. demonstrated that up regulation of drug efflux genes and increased efflux activity in persister cells of *E. coli* (Pu et al., 2016). Together with our results, these findings suggest that persister cells might be armed with various defense and offense factors that enable them to actively defend against environmental stresses before entering into deeper dormancy state. Thus, exploration of the gene expression profiles of persister cells will shed light on their surviving strategies in various host environments.

## Materials and methods

### Bacterial strains and plasmids

The bacterial strains used in this study are listed in Table S2. Bacteria were cultured in Luria–Bertani (LB) broth (10 g/l tryptone, 5 g/l Nacl, 5 g/l yeast extract, pH 7.0–7.5) or LB agar (LB broth containing 15 g/l agar) under aerobic conditions at 37°C. When needed, the medium was supplemented with tetracycline (100 μg/ml) (BBI life sciences, Shanghai, China), gentamicin (100 μg/ml) (BBI life sciences), streptomycin (50 μg/ml) (BBI life sciences), or carbenicillin (150 μg/ml) (BBI life sciences) for *P. aeruginosa*, and ampicillin (100 μg/ml) (BBI life sciences) for *E. coli*.

Plasmids used in this study are listed in Table S2. For DNA manipulation, standard protocols or manufacture instructions of commercial products were followed. Chromosomal gene mutations were generated as described previously (Hoang et al., 1998).

### Reverse transcription and quantitative RT PCR

Total RNA was isolated from bacteria at indicated time points with an RNeasy Minikit (Tiangen Biotech, Beijing, China). The cDNA was synthesized from total RNA using random primers and PrimeScript Reverse Transcriptase (TaKaRa, Dalian, China). Specific Primers (Table S2) were used for quantitative RT PCR. For quantitative RT PCR, cDNA was mixed with 4 pmol of forward and reverse primers and SYBR Premix Ex Taq™ II (TaKaRa) in a total reaction volume of 20 μl. The results were determined using a CFX Connect Real-Time system (Bio-Rad, USA).

### 5′ race analysis

The transcriptional start site of the *higB-higA* operon was determined by 5′ RACE (rapid amplification of cDNA ends). The cDNA was synthesized from total RNA using primer higA-R and higB-R. cDNA was purified with a DNA Clean kit (Sangon Biotech, Shanghai, China) and tailed with poly (dC) using terminal deoxynucleotidyl transferase (TaKaRa), then amplified by PCR. The obtained PCR product was ligated into a T-vector (TaKaRa), then sequenced.

### Electrophoretic mobility shift assay

Electrophoretic mobility shift assay (EMSA) was performed as described with minor modification (Sun et al., 2014). Briefly, a 38-bp DNA fragment corresponding to sequence up-stream of *higB* start codon or the 38-bp DNA fragment with palindrome sequence scrambled as a negative control was synthesized. DNA fragments (300 ng) were incubated with 0, 4 or 20 nM purified recombinant HigA protein at 30°C for 30 min in a 20-μl reaction (10 mM Tris-HCl, pH 7.6, 4% glycerol, 1 mM EDTA, 5 mM CaCl_2_, 100 mM NaCl, 10 mM-β-Mercaptoethanol). Samples were loaded onto a 8% native polyacrylamide gel in 0.5 × Tris-borate-EDTA (TBE) buffer (0.044 M Tris base, 0.044 M boric acid, 0.001 M EDTA, pH 8.0) that had been prerun for 1 h, electrophoresed on ice at 90 V for 2 h followed by DNA staining in 0.5 × TBE containing 0.5 μg/ml ethidium bromide. Bands were visualized with a molecular imager ChemiDoc™ XRS + (Bio-Rad).

### Antitoxin stabilization assays

Overnight culture of wild type PA14, a *clpP*::Tn or *lon*::Tn mutant harboring pMMB67EH-*higA*-His plasmid was sub-cultured in fresh LB broth to an OD_600_ of 0.5, then induced with 1 mM IPTG for 1 h, followed by treatment with 50 μg/ml streptomycin. Bacteria were collected at 0, 0.5, 1, 2, 3, 4, and 5 h, boiled in 1 × SDS loading buffer, then subjected to SDS-PAGE. Proteins was transferred onto a PVDF membrane and incubated with mouse anti-His antibody (1:2000) (Millipore, USA) at room temperature for 1 h. After washing with 1 × phosphate buffered saline (1 × PBS: 274 mM NaCl, 5.4 mM KCl, 20 mM Na_2_HPO_4_, 4 mM KH_2_PO_4_, pH 7.4) for four times, the membranes were incubated with a horseradish peroxidase-conjugated goat anti-mouse IgG (1:2000) (Promega, USA) at room temperature for 1 h. Signals were detected with the ECL-plus kit (Millipore) and visualized with a Bio-Rad molecular imager ChemiDoc™ XRS+.

### Persistence assay

Persistence of *P. aeruginosa* was measured by time-dependent killing experiments (Dörr et al., 2010). To test the persistence level induced by sublethal level of ciprofloxacin, overnight bacterial culture was sub-cultured in fresh LB broth and grown to an OD_600_ of 0.4 with or without 0.025 μg/ml ciprofloxacin. Then the bacterial cultures were exposed to 0.25 μg/ml ciprofloxacin. To test the effect of *higA* mutation or *higB* overexpression on persister formation, indicated strains were grown to an OD_600_ of 0.4, followed by treatment with 0.25 μg/ml ciprofloxacin. At indicated time points, the live bacterial number was determined by serial dilution and plating. The plate was incubated at 37°C for 24 h before colony counting.

### RNA sequencing and data analysis

PA14 and the *higA*::Tn mutant were cultured in LB broth at 37°C and harvested at log phage (OD_600_ of 0.8–1.0) and stationary phase (OD_600_ of 2.5–3.0). Total RNA was extracted with an RNeasy Protect Bacteria Mini Kit with on-column DNase I digestion (Qiagen, Shanghai, China). A Turbo DNA-free vigorous protocol was used for a second round of DNase treatment (Ambion). 16S, 23S, and 5S rRNA were removed using the Ribo-Zero Magnetic Kit (Bacteria) (Epicentre).

Gene expression analysis was conducted via Illumina RNA sequencing (RNA-Seq technology). RNA-Seq was conducted for 3 biological replicates of each sample. The rRNA-depleted RNA was fragmented to 150–200 bp in sizes, then first and second strand cDNA were synthesized, followed by end repair and adapter ligation. After 12 cycles of PCR enrichment, the quality of the libraries was assessed using a Bioanalyzer (Agilent Technologies). The libraries were sequenced using an Illumina HiSeq 2500 platform with a paired-end protocol and read lengths of 100-nt.

The sequencing data was analyzed using the method described previously (Chua et al., 2014). Sequence reads were mapped onto PA14 reference genome (NC_008463) using a CLC genomics Workbench 8.0 (CLC Bio-Qiagen, Aarhus, Denmark). The count data of expression values were then analyzed using a DESeq package of R/Bioconductor. The differentially expressed genes were identified by performing a negative binomial test using the DESeq, with the cut-off of fold-change larger than 2. The raw sequence reads were normalized by dividing with size factors, then Log_2_ (N + 1) transformed.

### Immunofluorescence assay

Bacteria with or without ciprofloxacin treatment were cytocentrifuged onto glass slides and fixed with 4% paraformaldehyde at room temperature for 30 min. Then bacteria were washed with 1 × PBS three times and permeabilized with 0.2% Triton X-100 in 1 × PBS at room temperature for 5 min. After washed twice with PBS, the bacteria were incubated with rabbit anti-PcrV serum (1:50) in PBSG (1 × PBS containing 0.1% gelatin) at 37°C for 1 h. The cells were washed twice with PBSG and incubated with the secondary antibody, green or red-conjugated goat anti- rabbit IgG (1:100) (Thermo Fisher Scientific, USA) in PBSG at 37°C for 1 h. To determine the viability, bacteria were stained with 1 μg/ml PI in 1 × PBS at room temperature for 15 min after 0.5 or 6 h ciprofloxacin treatment. Then cells were analyzed by a BX53 fluorescence microscope (Olympus, Japan).

### Cell culture and cytotoxicity assays

Raw264.7 cells and HeLa cells were cultured in DMEM medium with 10% fetal bovine serum (FBS) at 37°C in 5% CO_2_, and 95% air, supplemented with 1% penicillin/streptomycin and ciprofloxacin (10 μg/ml). Overnight bacterial culture was sub-cultured in fresh LB broth to OD_600_ of 0.8 before infection. Bacteria were washed once and resuspended in 1 × PBS. Raw264.7 and HeLa cells were infected with bacteria at a multiplicity of infection (MOI) of 10 or 40, respectively, in DMEM medium without FBS and antibiotics. At the end of incubation, lactate dehydrogenase (LDH) present in the supernatant was measured using the LDH cytotoxicity assay kit (Beyotime, Haimen, China). Cells treated with LDH release agent C0017-1 were used as a control of total release (100% LDH release). The background level (0% LDH release) was determined with DMEM medium. The percentage of cytotoxicity was calculated following the manufacturer's instruction.

### Effector delivery assay

HeLa cells were infected with strains containing P_*exoU*_*-exoU*-His at an MOI of 40. 1.5 h post infection, the cells were washed 3 times with 1 × PBS and lysed with 0.25% Trion-X 100. The Cell lysates were subjected to 10% SDS-PAGE. Proteins were transferred onto a PVDF membrane. The protein amounts of actin and ExoU were determined by Western blot analysis using mouse anti-His antibody or rabbit anti-β actin antibody (1:2000) (Cell Signaling Technology, USA).

### Protective effect of anti-PcrV antibody on Raw264.7 cells

Overnight bacterial cultures were sub-cultured in fresh LB broth to OD_600_ of 0.4 with 0.025 μg/ml ciprofloxacin, then the bacterial cultures were exposed to 0.125 μg/ml ciprofloxacin. Bacteria with or without ciprofloxacin treatment were washed with 1 × PBS, then added to 10^4^ Raw264.7 cells in 200 μl culture medium with various concentrations (0, 1:100, 1:1000) of either normal rabbit IgG or rabbit anti-PcrV IgG. Each mixture was incubated at 37°C for 3.5 h. Cytotoxicity was measured by LDH release assay as described above.

## Author contributions

Conceived and designed the experiments: WW, ML, SJ, ZC. Performed the experiments: ML, YuL, YiL, JS, RC, LZ, YJ, LY, YaL. Analyzed the data: ML, WW, ZC, SJ, FB, LY, YaL. Wrote the paper: ML, WW, ZC, SJ.

## Funding

This work was supported by National Science Foundation of China (31670130, 31370168 and 31370167); Program of international S&T cooperation (2015DFG32500) and Science and Technology Committee of Tianjin (15JCYBJC53900 and 15JCZDJC33000). The funders had no role in study design, data collection and interpretation, or the decision to submit the work for publication.

### Conflict of interest statement

The authors declare that the research was conducted in the absence of any commercial or financial relationships that could be construed as a potential conflict of interest.

## Supplementary material

Figure S1 **Expression levels of T3SS genes in wild type PA14, a *higA*::Tn mutant and a complemented strain. (A)** Cleavage of HigA by the Lon protease. Wild type PA14, the *clpP*::Tn and *lon*::Tn mutants carrying pMMB67EH-*higA*-His were cultured in the presence of 1 mM IPTG for 1 h. Then 50 μg/ml spectinomycin was added to the medium. At indicated time points, the HigA-His levels were determined by Western blot analysis with an anti-His antibody. Density of each band was quantified with Image J. This graph represents the results of three independent experiments.**(B)** Relative mRNA levels of *exsC, exsA, pcrV* and *exoU* in indicated strains at stationary growth phase (OD_600_ = 2.5~3.0). Data represents the mean ± standard deviation from three independent experiments performed in triplicates. ^*^*p* < 0.05, ^**^*p* < 0.01, ^***^*p* < 0.005 compared to wild type PA14 by Student's *t*-test. **(C)** Fluorescence microscopy of PA14 and the *higA*::Tn mutant containing P_*higB*_*-mcherry*. Bacteria were grown in LB to an OD_600_ of 3.0, collected and washed with PBS twice. The bacteria were fixed, permeabilized and then stained with rabbit anti-PcrV followed by Alex Fluor 594–labeled goat anti–rabbit immunoglobulin. Bar = 20 μm. **(D)** Bacteria carrying an *exoU*-His driven by its native promoter (P_*exoU*_*-exoU*-His) were grown in LB at 37°C. At stationary growth phase, bacteria were collected. Samples from equivalent bacterial cells were loaded into SDS-PAGE gels and stained with Coomassie blue or probed with an anti-His antibody. **(E)** HeLa cells were infected with indicated strains at an MOI of 40 for 3 h, followed by the LDH release assay. The values and bars represent the means and standard deviations of triplicate measurements. ^*^*p* < 0.05 by Student's *t*-test. HeLa cells were infected with strains containing P_*exoU*_*-exoU*-His **(F)** or P_*lac*_-*gfp*-His **(G)** at an MOI of 40. 1.5 h after infection, the cells were washed 3 times with PBS and lysed with 0.25% Trion-X 100. The intracellular levels of ExoU-His and GFP-His were determined by Western blot analysis.

Figure S2 **HigB promotes expression levels of T3SS genes and cytotoxicity. (A)** Relative mRNA levels of T3SS genes in wild type PA14, the *higA*::Tn and Δ*higB*Δ*higA* mutants. Bacteria were grown to an OD_600_ of 1.0, followed by total RNA isolation. The mRNA levels of *exsC, exsA, pcrV*, and *exoU* were determined by quantitative RT-PCR. **(B)** Raw264.7 cell cells were infected with indicated strains at an MOI of 10 for 3.5 h. The relative cytotoxicity was determined by the LDH release assay. **(C)** PA14 or the *exsA*::Tn mutant containing pMMB67EH-*higB*-His or pMMB67EH was grown in the presence of indicated concentrations of IPTG for 3 h. The levels of HigB in bacterial cell lysates were determined by Western blot analysis. The loading control was displayed in Figure 4E. The relative density of each band was determined by Image J. **(D)** HeLa cells were infected with the indicated bacteria at an MOI of 40 for 3 h, followed by LDH release assay. ND, not detectable. The values and bars represent the means and standard deviations of triplicate measurements. ^*^*p* < 0.05, ^**^*p* < 0.01, ^***^*p* < 0.005 by Student's *t*-test.

Figure S3 **Fluorescence microscopy of PA14 containing P_*higB*_*-gfp***. At an OD_600_ of 0.3, bacteria were incubated with 0.025 μg/ml ciprofloxacin for 2 h and then treated with 0.25 μg/ml ciprofloxacin for 6 h in LB. The ciprofloxacin treated and untreated bacteria were collected and washed with PBS twice. The bacterial cells were stained with PI **(A)** or immunostained with rabbit anti-PcrV followed by Alex Fluor 594–labeled goat anti–rabbit immunoglobulin. Bar = 10 μm **(B)**. Quantification of fluorescence positive cells was based on analysis of about 100 cells from three different samples. **(C)** PA14 or the *exsA*::Tn mutant were cultured in the presence or absence of 0.025 μg/ml ciprofloxacin for 2 h and then treated with 0.125 μg/ml ciprofloxacin for 30 min or 6 h. Or, the PA14 cells were incubated at 50°C for 30 min. Live bacteria were collected. Raw264.7 cells were infected with the indicated bacteria at an MOI of 10 for 3.5 h. **(D)** HeLa cells were infected with the indicated bacteria at MOI of 40 for 3.0 h. The relative cytotoxicity levels were determined by LDH release assay. ND, not detectable. ^*^*p* < 0.05, compared to each of the other samples by Student's *t*-test.

Figure S4 **Fluorescence microscopy of Δ*higB* mutant containing P_*higB*_*-gfp***. At an OD_600_ of 0.3, a Δ*higB* mutant containing P_*higB*_*-gfp* were incubated with 0.025 μg/ml ciprofloxacin for 2 h and then treated with 0.125 μg/ml ciprofloxacin for 30 min in LB. The ciprofloxacin treated and untreated bacteria were collected and washed with PBS twice. The bacterial cells were stained with PI **(A)** or immunostained with rabbit anti-PcrV followed by Alex Fluor 594–labeled goat anti–rabbit immunoglobulin **(B)**. Bar = 10 μm.

Figure S5 **Fluorescence microscopy of Δ*higB*Δ*higA* mutant containing P_*higB*_*-gfp***. At the OD_600_ of 0.3, a Δ*higB*Δ*higA* mutant containing P_*higB*_*-gfp* were incubated with 0.025 μg/ml ciprofloxacin for 2 h and then treated with 0.125 μg/ml ciprofloxacin for 30 min in LB. The ciprofloxacin treated and untreated bacteria were collected and washed with PBS twice. The bacterial cells were stained with PI **(A)** or immunostained with rabbit anti-PcrV followed by Alex Fluor 594–labeled goat anti–rabbit immunoglobulin **(B)**. Bar = 10 μm.

Table S1 **PA14 Transcriptome analysis: differentially regulated genes**.

Table S2 **Bacterial strains, plasmids and primers used in this study**.
