# Supplementary figures and images for: HigB of Pseudomonas aeruginosa Enhances Killing of Phagocytes by Up-Regulating the Type III Secretion System in Ciprofloxacin Induced Persister Cells
Source: Front Cell Infect Microbiol. 2016 Oct 14;6:125. doi: 10.3389/fcimb.2016.00125 (PMC5064212; doi:10.3389/fcimb.2016.00125)

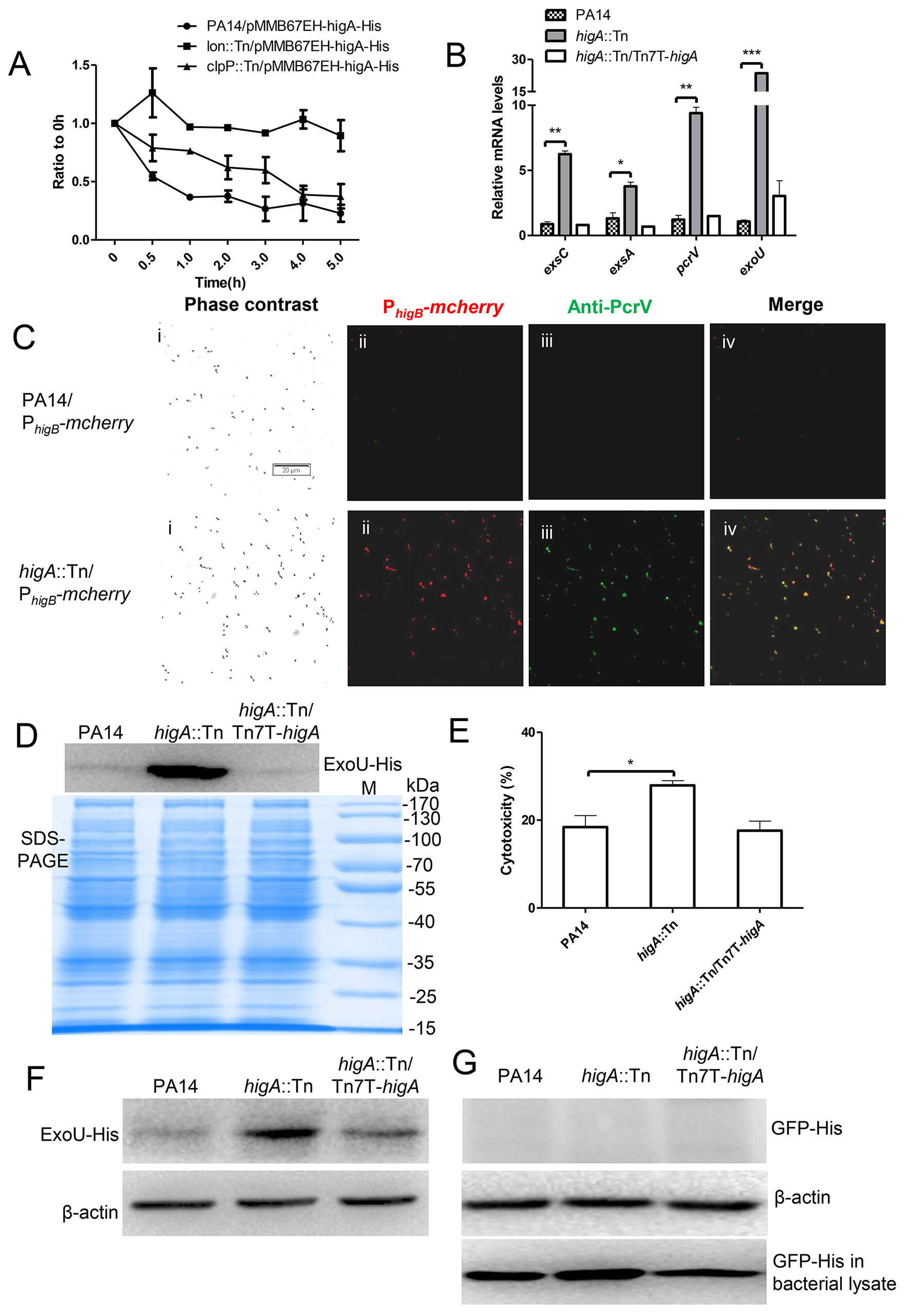

Supplement: Figure S1 — Expression levels of T3SS genes in wild type PA14, a higA::Tn mutant and a complemented strain. (A) Cleavage of HigA by the Lon protease. Wild type PA14, the clpP::Tn and lon::Tn mutants carrying pMMB67EH-higA-His were cultured in the presence of 1 mM IPTG for 1 h. Then 50 μg/ml spectinomycin was added to the medium. At indicated time points, the HigA-His levels were determined by Western blot analysis with an anti-His antibody. Density of each band was quantified with Image J. This graph represents the results of three independent experiments.(B) Relative mRNA levels of exsC, exsA, pcrV and exoU in indicated strains at stationary growth phase (OD600 = 2.5~3.0). Data represents the mean ± standard deviation from three independent experiments performed in triplicates. *p < 0.05, **p < 0.01, ***p < 0.005 compared to wild type PA14 by Student's t-test. (C) Fluorescence microscopy of PA14 and the higA::Tn mutant containing PhigB-mcherry. Bacteria were grown in LB to an OD600 of 3.0, collected and washed with PBS twice. The bacteria were fixed, permeabilized and then stained with rabbit anti-PcrV followed by Alex Fluor 594–labeled goat anti–rabbit immunoglobulin. Bar = 20 μm. (D) Bacteria carrying an exoU-His driven by its native promoter (PexoU-exoU-His) were grown in LB at 37°C. At stationary growth phase, bacteria were collected. Samples from equivalent bacterial cells were loaded into SDS-PAGE gels and stained with Coomassie blue or probed with an anti-His antibody. (E) HeLa cells were infected with indicated strains at an MOI of 40 for 3 h, followed by the LDH release assay. The values and bars represent the means and standard deviations of triplicate measurements. *p < 0.05 by Student's t-test. HeLa cells were infected with strains containing PexoU-exoU-His (F) or Plac-gfp-His (G) at an MOI of 40. 1.5 h after infection, the cells were washed 3 times with PBS and lysed with 0.25% Trion-X 100. The intracellular levels of ExoU-His and GFP-His were determined by Wester [file Image1.TIF]

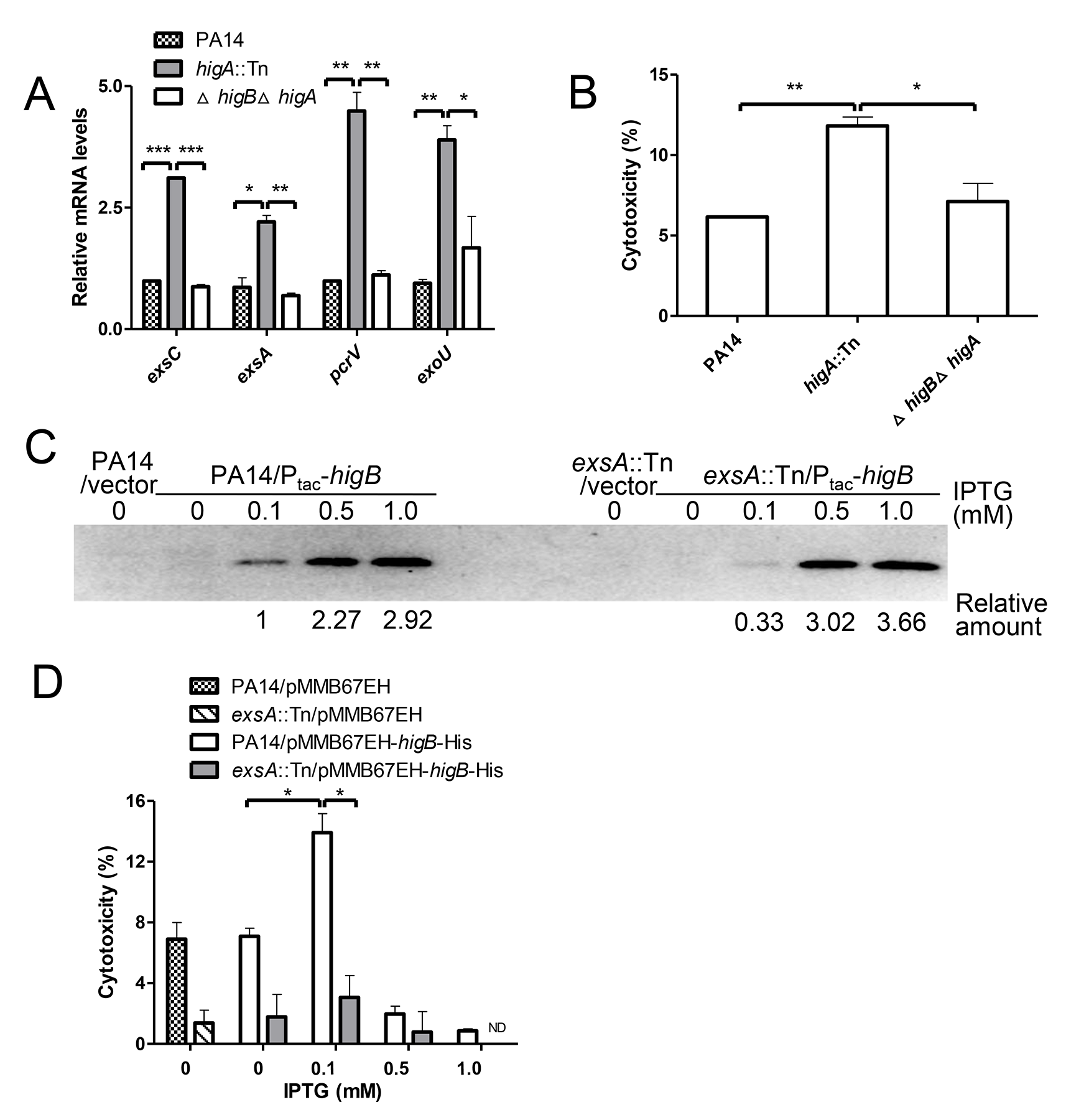

Supplement: Figure S2 — HigB promotes expression levels of T3SS genes and cytotoxicity. (A) Relative mRNA levels of T3SS genes in wild type PA14, the higA::Tn and ΔhigBΔhigA mutants. Bacteria were grown to an OD600 of 1.0, followed by total RNA isolation. The mRNA levels of exsC, exsA, pcrV, and exoU were determined by quantitative RT-PCR. (B) Raw264.7 cell cells were infected with indicated strains at an MOI of 10 for 3.5 h. The relative cytotoxicity was determined by the LDH release assay. (C) PA14 or the exsA::Tn mutant containing pMMB67EH-higB-His or pMMB67EH was grown in the presence of indicated concentrations of IPTG for 3 h. The levels of HigB in bacterial cell lysates were determined by Western blot analysis. The loading control was displayed in Figure 4E. The relative density of each band was determined by Image J. (D) HeLa cells were infected with the indicated bacteria at an MOI of 40 for 3 h, followed by LDH release assay. ND, not detectable. The values and bars represent the means and standard deviations of triplicate measurements. *p < 0.05, **p < 0.01, ***p < 0.005 by Student's t-test. [file Image2.TIF]

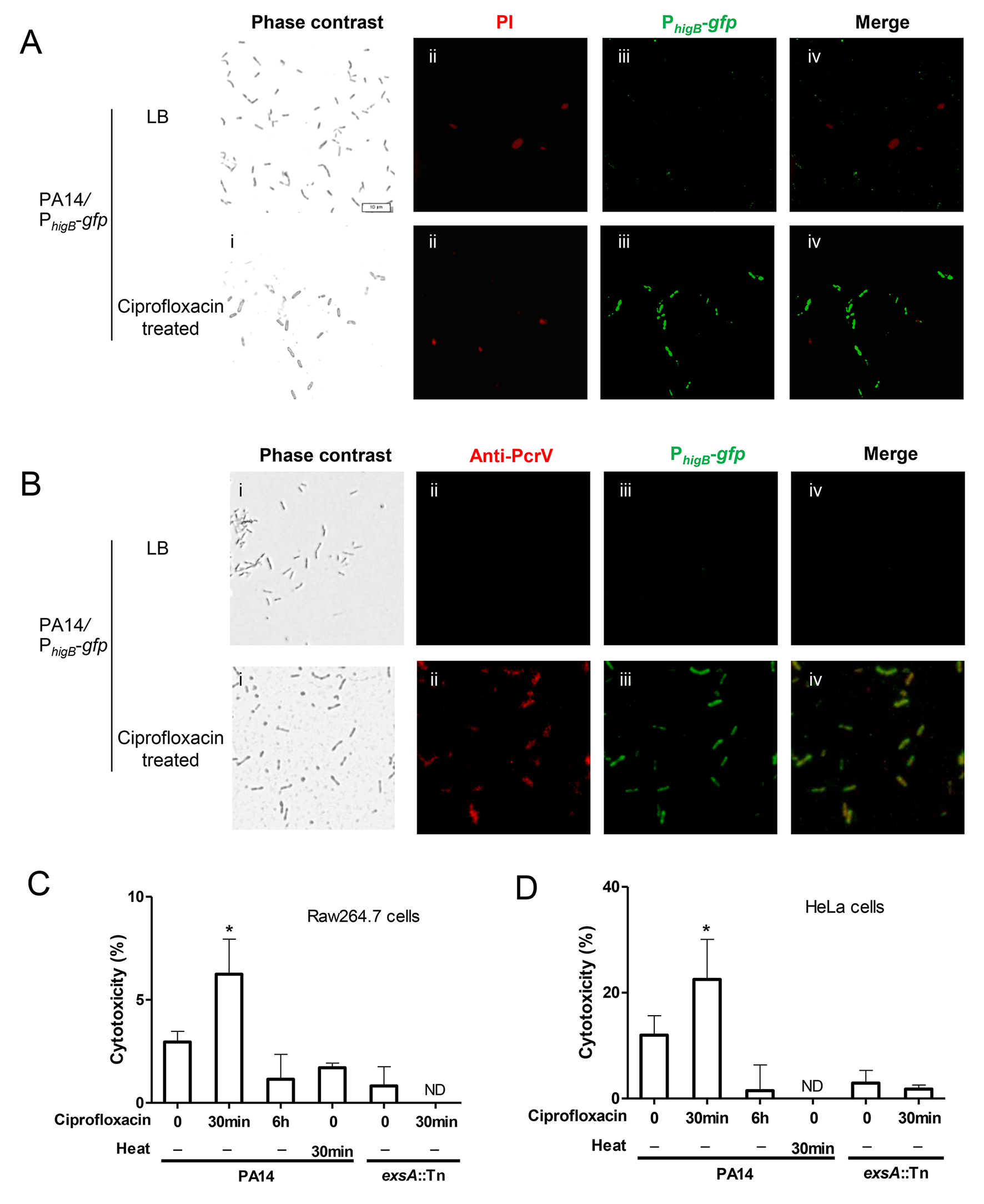

Supplement: Figure S3 — Fluorescence microscopy of PA14 containing PhigB-gfp. At an OD600 of 0.3, bacteria were incubated with 0.025 μg/ml ciprofloxacin for 2 h and then treated with 0.25 μg/ml ciprofloxacin for 6 h in LB. The ciprofloxacin treated and untreated bacteria were collected and washed with PBS twice. The bacterial cells were stained with PI (A) or immunostained with rabbit anti-PcrV followed by Alex Fluor 594–labeled goat anti–rabbit immunoglobulin. Bar = 10 μm (B). Quantification of fluorescence positive cells was based on analysis of about 100 cells from three different samples. (C) PA14 or the exsA::Tn mutant were cultured in the presence or absence of 0.025 μg/ml ciprofloxacin for 2 h and then treated with 0.125 μg/ml ciprofloxacin for 30 min or 6 h. Or, the PA14 cells were incubated at 50°C for 30 min. Live bacteria were collected. Raw264.7 cells were infected with the indicated bacteria at an MOI of 10 for 3.5 h. (D) HeLa cells were infected with the indicated bacteria at MOI of 40 for 3.0 h. The relative cytotoxicity levels were determined by LDH release assay. ND, not detectable. *p < 0.05, compared to each of the other samples by Student's t-test. [file Image3.TIF]

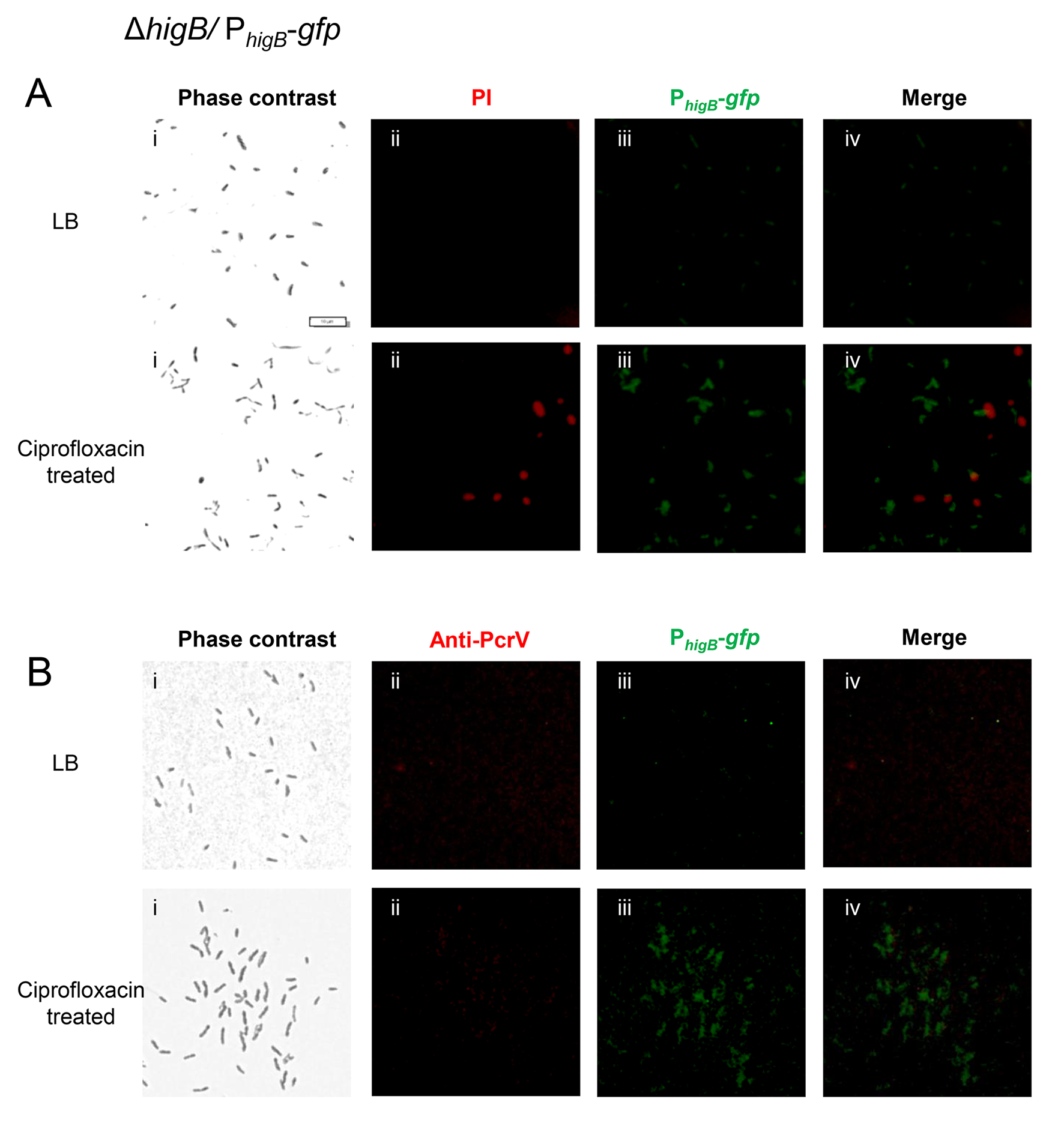

Supplement: Figure S4 — Fluorescence microscopy of ΔhigB mutant containing PhigB-gfp. At an OD600 of 0.3, a ΔhigB mutant containing PhigB-gfp were incubated with 0.025 μg/ml ciprofloxacin for 2 h and then treated with 0.125 μg/ml ciprofloxacin for 30 min in LB. The ciprofloxacin treated and untreated bacteria were collected and washed with PBS twice. The bacterial cells were stained with PI (A) or immunostained with rabbit anti-PcrV followed by Alex Fluor 594–labeled goat anti–rabbit immunoglobulin (B). Bar = 10 μm. [file Image4.TIF]

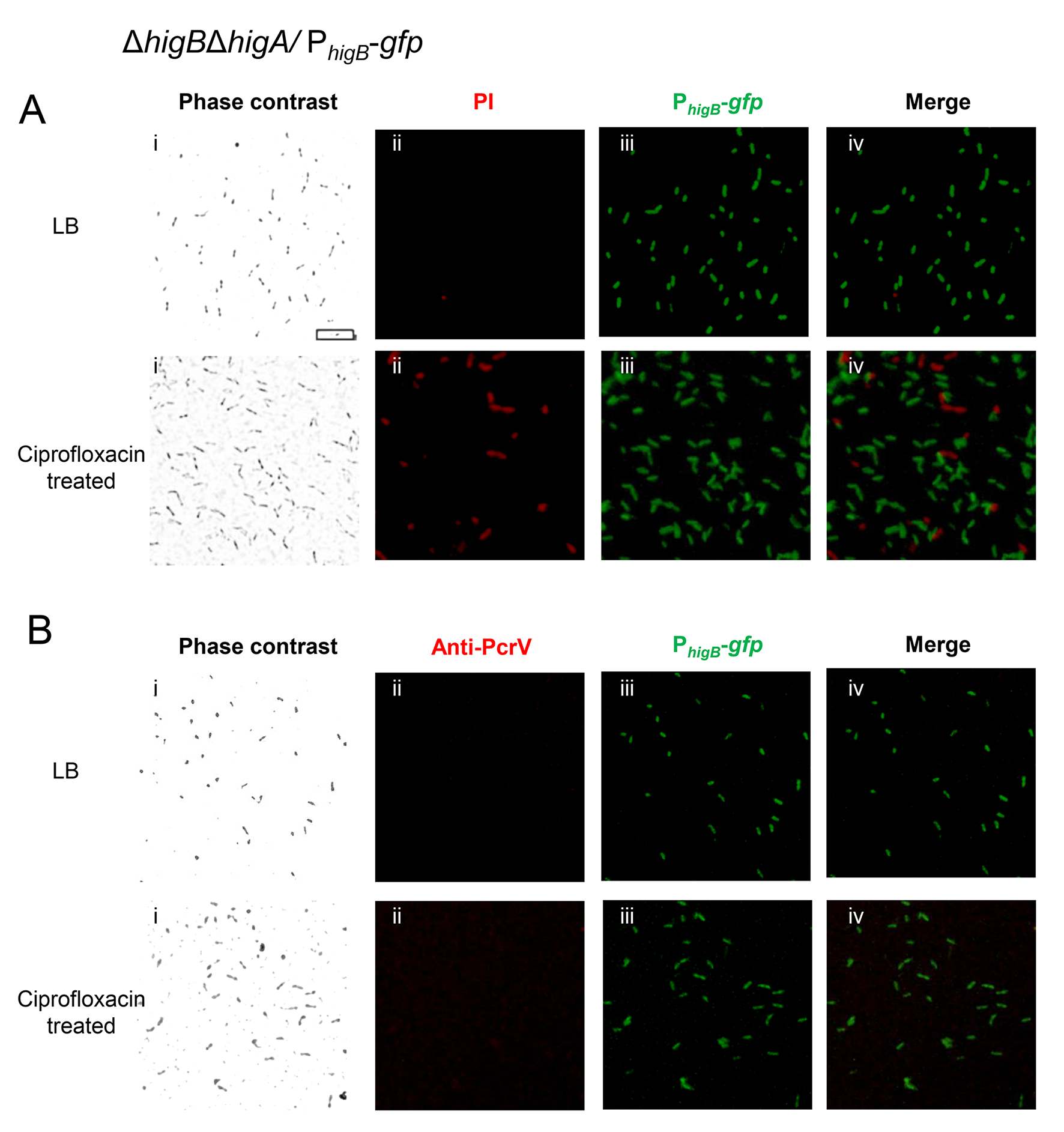

Supplement: Figure S5 — Fluorescence microscopy of ΔhigBΔhigA mutant containing PhigB-gfp. At the OD600 of 0.3, a ΔhigBΔhigA mutant containing PhigB-gfp were incubated with 0.025 μg/ml ciprofloxacin for 2 h and then treated with 0.125 μg/ml ciprofloxacin for 30 min in LB. The ciprofloxacin treated and untreated bacteria were collected and washed with PBS twice. The bacterial cells were stained with PI (A) or immunostained with rabbit anti-PcrV followed by Alex Fluor 594–labeled goat anti–rabbit immunoglobulin (B). Bar = 10 μm. [file Image5.TIF]
