# Supplementary material for: HigB of Pseudomonas aeruginosa Enhances Killing of Phagocytes by Up-Regulating the Type III Secretion System in Ciprofloxacin Induced Persister Cells
Source: Front Cell Infect Microbiol. 2016 Oct 14;6:125. doi: 10.3389/fcimb.2016.00125 (PMC5064212; doi:10.3389/fcimb.2016.00125)
Supplement: Table S1 — PA14 Transcriptome analysis: differentially regulated genes. [file Table1.DOC]

Table S1. PA14 Transcriptome analysis: differentially regulated genes

| **Locus Tag PA14** | **Locus Tag PAO1** | **Name** | **Product** | **Fold changes *higA*::Tn/WT (E)** | **P value** | **Fold changes *higA*::Tn/WT (S)** | **P value** |
| --- | --- | --- | --- | --- | --- | --- | --- |
| PA14_RS00230 | PA0044 | *exoT* | exoenzyme T | 6.14094447 | 4.014E-28 | 8.189608036 | 3.39E-41 |
| PA14_RS00705 | PA0139 | *ahpC* | alkyl hydroperoxide reductase | 0.291802619 | 0.1590083 | 0.13418736 | 0.004927 |
| PA14_RS00715 | PA0141 |  | hypothetical protein | 0.358050632 | 5.756E-06 | 0.299162182 | 1.27E-06 |
| PA14_RS00915 | PA0179 |  | two-component response regulator | 0.289322981 | 0.0037789 | 0.436066456 | 0.072733 |
| PA14_RS01025 | PA0208 | *mdcA* | malonate decarboxylase subunit alpha | 3.508996838 | 1.644E-08 | 0.418137808 | 8.54E-05 |
| PA14_RS01035 | PA0210 | *mdcC* | malonate decarboxylase subunit delta | 0.329192438 | 0.3575345 | 0.1890651 | 0.012159 |
| PA14_RS01045 | PA0212 | *mdcE* | malonate decarboxylase subunit gamma | 2.12000891 | 0.6336017 | 0.348377117 | 0.083398 |
| PA14_RS02350 | PA0446 |  | hypothetical protein | 0.491258848 | 0.0821863 | 0.301637059 | 0.000418 |
| PA14_RS02355 | PA0447 | *gcdH* | glutaryl-CoA dehydrogenase | 0.44857259 | 0.2602431 | 0.199147686 | 0.004532 |
| PA14_RS02365 | PA0449 |  | hypothetical protein | 0.365928076 | 0.3539245 | 0.200160694 | 0.045079 |
| PA14_RS02415 | PA0459 |  | ClpA/B protease ATP binding subunit | 0.208445685 | 2.375E-09 | 0.45351025 | 0.026585 |
| PA14_RS02680 | PA0513 | *nirG* | transcriptional regulator | 0.311720281 | 0.000428 | 0.488905157 | 0.170944 |
| PA14_RS02690 | PA0515 |  | transcriptional regulator | 0.353904442 | 0.0002212 | 0.484390737 | 0.035073 |
| PA14_RS02695 | PA0516 | *nirF* | heme d1 biosynthesis protein NirF | 0.362826473 | 0.0152909 | 0.306274454 | 0.023879 |
| PA14_RS02710 | PA0519 | *nirS* | nitrite reductase | 0.417364315 | 0.3190753 | 0.083666994 | 0.015207 |
| PA14_RS03065 | PA0587 |  | hypothetical protein | 0.434770099 | 3.288E-06 | 0.463195133 | 7.69E-06 |
| PA14_RS03220 | PA0618 |  | phage baseplate assembly protein | 0.409036594 | 6.206E-06 | 0.48669741 | 0.003034 |
| PA14_RS03230 | PA0620 |  | tail fiber protein | 0.438472034 | 2.774E-07 | 0.438807424 | 5.61E-06 |
| PA14_RS03235 | PA0621 |  | tail fiber assembly protein | 0.371540279 | 1.552E-06 | 0.419733197 | 0.000174 |
| PA14_RS03240 | PA0622 |  | phage tail sheath protein | 0.341177803 | 7.81E-12 | 0.410235622 | 3.22E-07 |
| PA14_RS03245 | PA0623 |  | phage tail tube protein | 0.295474283 | 2.795E-14 | 0.340702257 | 3.77E-10 |
| PA14_RS03250 | PA0624 |  | hypothetical protein | 0.333436937 | 1.735E-10 | 0.380205778 | 6.16E-08 |
| PA14_RS03255 | PA0625 |  | tail length determinator protein | 0.408280703 | 4.046E-07 | 0.444209517 | 9.29E-05 |
| PA14_RS03260 | PA0626 |  | hypothetical protein | 0.447340645 | 0.0012253 | 0.497432314 | 0.020354 |
| PA14_RS03270 | PA0628 |  | phage late control gene D protein | 0.438121591 | 0.0001148 | 0.497011719 | 0.002898 |
| PA14_RS03275 | PA0629 |  | lytic enzyme | 0.440421911 | 0.0005059 | 0.455777972 | 0.001613 |
| PA14_RS03295 | PA0634 |  | hypothetical protein | 0.342432837 | 7.653E-11 | 0.370801151 | 1.18E-08 |
| PA14_RS03300 | PA0635 |  | hypothetical protein | 0.350342863 | 1.769E-09 | 0.365176247 | 9.65E-08 |
| PA14_RS03305 | PA0636 |  | hypothetical protein | 0.417618073 | 1.331E-07 | 0.461181483 | 4.79E-05 |
| PA14_RS03315 | PA0638 |  | minor tail protein L | 0.427882578 | 2.786E-07 | 0.467057408 | 1.88E-05 |
| PA14_RS03320 | PA0639 |  | hypothetical protein | 0.458626849 | 0.0019186 | 0.496485586 | 0.018289 |
| PA14_RS03325 | PA0640 |  | bacteriophage protein | 0.437763577 | 4.198E-05 | 0.449467405 | 0.000229 |
| PA14_RS03330 | PA0641 |  | phage-related protein, tail component | 0.477517077 | 0.0001232 | 0.489560528 | 0.000727 |
| PA14_RS22455 | PA0713 |  | hypothetical protein | 0.357617389 | 2.026E-05 | 0.245644697 | 4.78E-06 |
| PA14_RS19875 | PA0723 | *coaB* | coat protein B of bacteriophage Pf1) | 0.487699619 | 0.0055447 | 2.211982009 | 8.83E-05 |
| PA14_RS21790 | PA0830 |  | hypothetical protein | 0.452037652 | 0.007764 | 0.239995252 | 2.98E-08 |
| PA14_RS21095 | PA0962 |  | DNA-binding stress protein | 0.372947851 | 0.0220793 | 0.252533526 | 0.00044 |
| PA14_RS20990 | PA0981 |  | hypothetical protein | 0.448313059 | 3.337E-05 | 0.389252859 | 3.04E-06 |
| PA14_RS24170 | PA0985 |  | pyocin S5 | 0.214825318 | 9.867E-17 | 0.394454288 | 2.7E-06 |
| PA14_RS20350 | PA1104 | *fliI* | flagellum-specific ATP synthase | 9.250179933 | 2.996E-40 | 11.72411053 | 1.25E-49 |
| PA14_RS20190 | PA1134 |  | hypothetical protein | 2.561541943 | 0.1781374 | 5.124705228 | 0.000925 |
| PA14_RS19535 | PA1249 | *aprA* | alkaline metalloproteinase | 0.16157477 | 2.51E-14 | 0.074929612 | 1.4E-24 |
| PA14_RS19090 | PA1337 | *ansB* | glutaminase-asparaginase | 0.440441384 | 0.0111153 | 0.483528624 | 0.03682 |
| PA14_RS18025 | PA1555 |  | cytochrome c oxidase, cbb3-type subunit III | 0.310056038 | 8.605E-05 | 0.269544738 | 0.000221 |
| PA14_RS18015 | PA1556 |  | cbb3-type cytochrome c oxidase subunit II | 0.342110423 | 6.052E-05 | 0.238375873 | 2.48E-06 |
| PA14_RS18010 | PA1557 |  | cbb3-type cytochrome c oxidase subunit I | 0.482581943 | 0.0427928 | 0.446666738 | 0.041287 |
| PA14_RS17815 | PA1596 | *htpG* | heat shock protein 90 | 0.242586325 | 0.0049954 | 0.455640656 | 0.106681 |
| PA14_RS17315 | PA1690 | *pscU* | translocation protein in type III secretion | 5.039503968 | 5.032E-18 | 3.733181364 | 1.51E-12 |
| PA14_RS17310 | PA1691 | *pscT* | translocation protein in type III secretion | 6.568684886 | 5.634E-16 | 3.900571154 | 2.57E-08 |
| PA14_RS17305 | PA1692 |  | translocation protein in type III secretion | 4.021650979 | 3.452E-05 | 5.279984166 | 4.73E-06 |
| PA14_RS17300 | PA1693 | *pscR* | type III secretion system protein | 4.259240157 | 2.162E-13 | 4.455856488 | 6.79E-11 |
| PA14_RS17295 | PA1694 | *pscQ* | type III secretion system protein | 4.939899745 | 9.638E-21 | 5.340780533 | 1.3E-21 |
| PA14_RS17285 | PA1696 | *pscO* | translocation protein in type III secretion | 6.773003747 | 4.838E-13 | 7.603388941 | 1.83E-16 |
| PA14_RS17275 | PA1698 | *popN* | Type III secretion outer membrane protein PopN precursor | 9.668090908 | 1.988E-17 | 10.30346371 | 2.25E-19 |
| PA14_RS17270 | PA1699 |  | protein in type III secretion | 7.646267085 | 6.87E-22 | 10.89093389 | 3.05E-29 |
| PA14_RS17265 | PA1700 |  | type III secretion protein | 9.520440847 | 3.653E-12 | 8.711029831 | 1.06E-11 |
| PA14_RS17260 | PA1701 |  | hypothetical protein | 15.06928042 | 3.345E-26 | 4.898149437 | 6.08E-11 |
| PA14_RS17255 | PA1702 |  | hypothetical protein | 7.637331138 | 1.402E-07 | 3.460824847 | 0.005321 |
| PA14_RS17250 | PA1703 | *pcrD* | type III secretory apparatus protein PcrD | 8.296437422 | 2.545E-36 | 4.418824195 | 8.01E-21 |
| PA14_RS17245 | PA1704 | *pcrR* | transcriptional regulator protein PcrR | 10.17979896 | 1.097E-07 | 3.672822006 | 0.001038 |
| PA14_RS17240 | PA1705 | *pcrG* | regulator in type III secretion | 10.21045719 | 4.383E-09 | 7.619511672 | 4.03E-08 |
| PA14_RS17235 | PA1706 | *pcrV* | type III secretion protein PcrV | 6.118342715 | 4.345E-30 | 9.030041987 | 5.9E-44 |
| PA14_RS17230 | PA1707 | *pcrH* | regulatory protein PcrH | 6.340626212 | 9.183E-29 | 19.66204987 | 1.89E-73 |
| PA14_RS17225 | PA1708 | *popB* | translocator protein PopB | 5.914447239 | 1.39E-21 | 10.59291018 | 1.1E-37 |
| PA14_RS17220 | PA1709 | *popD* | translocator outer membrane protein PopD precursor | 4.515915406 | 3.142E-12 | 6.589804671 | 5.54E-20 |
| PA14_RS17215 | PA1710 | *exsC* | exoenzyme S synthesis protein C | 4.806813446 | 1.563E-23 | 6.655236655 | 3.21E-34 |
| PA14_RS17210 | PA1711 |  | hypothetical protein | 4.700100895 | 2.32E-05 | 5.277074344 | 1.48E-06 |
| PA14_RS17205 | PA1712 | *exsB* | exoenzyme S synthesis protein B | 5.985476838 | 1.565E-21 | 5.143797255 | 5.97E-22 |
| PA14_RS17200 | PA1713 | *exsA* | transcriptional regulator ExsA | 5.374177295 | 4.245E-24 | 4.823894813 | 4.09E-22 |
| PA14_RS17195 | PA1714 |  | hypothetical protein | 6.886451015 | 2.26E-16 | 9.658477637 | 9.68E-24 |
| PA14_RS17190 | PA1715 | *pscB* | type III export apparatus protein | 10.82273635 | 2.913E-27 | 7.750285742 | 1.25E-22 |
| PA14_RS17185 | PA1716 | *pscC* | Type III secretion outer membrane protein PscC precursor | 7.544784211 | 8.131E-34 | 5.668366355 | 5.06E-26 |
| PA14_RS17180 | PA1717 | *pscD* | type III export protein PscD | 8.175959723 | 8.092E-17 | 6.117952422 | 5.71E-14 |
| PA14_RS17175 | PA1718 | *pscE* | type III export protein PscE | 3.671123106 | 3.435E-10 | 5.666252879 | 1.5E-18 |
| PA14_RS17170 | PA1719 | *pscF* | type III export protein PscF | 3.485661993 | 4.185E-07 | 6.825171541 | 4.2E-14 |
| PA14_RS17165 | PA1720 | *pscG* | type III export protein PscG | 4.786675009 | 3.267E-17 | 6.693601748 | 4.94E-24 |
| PA14_RS17160 | PA1721 | *pscH* | type III export protein PscH | 5.776113759 | 2.076E-19 | 5.983977658 | 2.64E-19 |
| PA14_RS17155 | PA1722 | *pscI* | type III export protein PscI | 5.716672184 | 5.44E-22 | 4.898014131 | 2.78E-19 |
| PA14_RS17150 | PA1723 | *pscJ* | pscJ type III export protein | 6.077783835 | 7.628E-27 | 5.099676258 | 1.98E-22 |
| PA14_RS17145 | PA1724 | *pscK* | PscK type III export protein | 10.68638134 | 3.18E-32 | 5.261505115 | 2E-14 |
| PA14_RS17140 | PA1725 | *pscL* | type III secretion system protein | 7.366724651 | 4.459E-30 | 5.8157518 | 2.25E-22 |
| PA14_RS17030 | PA1746 |  | hypothetical protein | 0.453440496 | 0.0001216 | 0.181272249 | 1.1E-13 |
| PA14_RS17025 | PA1747 |  | hypothetical protein | 0.489914276 | 0.1647634 | 0.271550545 | 0.022236 |
| PA14_RS16190 | PA1900 | *phzB2* | phenazine biosynthesis protein | 0.19737304 | 0.0011965 | 0.40879835 | 0.095594 |
| PA14_RS16185 | PA1901;PA4212 | *phzC2* | phenazine biosynthesis protein PhzC | 0.328121824 | 0.004075 | 0.393726433 | 0.00652 |
| PA14_RS03830 | PA1902;PA4213 | *phzD1* | phenazine biosynthesis protein PhzD | 0.290734255 | 0.0411354 | 0.46942338 | 0.055494 |
| PA14_RS15705 | PA1999 |  | CoA transferase, subunit A | 0.186628896 | 2.361E-07 | 3.158916891 | 0.000212 |
| PA14_RS15700 | PA2000 |  | CoA transferase subunit B | 0.174588725 | 4.147E-06 | 2.419374902 | 0.023192 |
| PA14_RS15185 | PA2110 |  | hypothetical protein | 3.203683391 | 3.285E-08 | 2.17802407 | 7.51E-05 |
| PA14_RS15175 | PA2112 |  | LamB/YcsF family protein | 2.609272557 | 4.695E-05 | 2.217709239 | 0.001881 |
| PA14_RS15170 | PA2113 |  | porin | 2.252820656 | 5.098E-05 | 2.031969991 | 0.000417 |
| PA14_RS15165 | PA2114 |  | major facilitator transporter | 3.372629149 | 8.079E-07 | 3.920001724 | 4.88E-09 |
| PA14_RS15155 | PA2116 |  | hypothetical protein | 2.157943105 | 3.35E-06 | 4.432495472 | 4.65E-22 |
| PA14_RS14985 | PA2146 |  | hypothetical protein | 0.465408328 | 0.4161285 | 0.14222493 | 0.008822 |
| PA14_RS14940 | PA2154 |  | hypothetical protein | 2.036005066 | 0.4399226 | 0.425907017 | 2.14E-05 |
| PA14_RS14930 | PA2156 |  | hypothetical protein | 4.470657645 | 0.0121089 | 0.412699776 | 0.000579 |
| PA14_RS14925 | PA2157 |  | hypothetical protein | 2.812242719 | 0.0896827 | 0.419453238 | 1.12E-05 |
| PA14_RS14915 | PA2159 |  | hypothetical protein | 2.603953648 | 0.4947393 | 0.36384829 | 0.00039 |
| PA14_RS14905 | PA2161 |  | hypothetical protein | 3.155435777 | 1 | 0.278461654 | 0.012294 |
| PA14_RS14900 | PA2162 |  | maltooligosyl trehalose synthase | 2.369924178 | 0.0154213 | 0.354005467 | 8.67E-10 |
| PA14_RS14895 | PA2163 |  | 4-alpha-glucanotransferase | 2.367349605 | 0.2954026 | 0.399175734 | 8.96E-05 |
| PA14_RS14850 | PA2173 |  | hypothetical protein | 0.257462448 | 0.2077993 | 0.162455265 | 3.2E-09 |
| PA14_RS14810 | PA2179 |  | hypothetical protein | 3.122123942 | 0.0036372 | 0.432589199 | 0.000744 |
| PA14_RS14795 | PA2187 |  | hypothetical protein | 5.224206764 | 4.86E-17 | 3.660931745 | 4.71E-14 |
| PA14_RS14790 | PA2189 |  | hypothetical protein | 5.812343355 | 3.842E-23 | 4.856424564 | 5.34E-23 |
| PA14_RS14785 | PA2191 | *exoY* | adenylate cyclase | 6.588517796 | 1.354E-30 | 6.052621003 | 1.75E-31 |
| PA14_RS14775 | PA2194 | *hcnB* | hydrogen cyanide synthase HcnB | 0.377271221 | 7.071E-10 | 2.053709726 | 2.37E-05 |
| PA14_RS13795 | PA2384 |  | hypothetical protein | 3.656788106 | 0.5201014 | 45.52761432 | 0.033451 |
| PA14_RS13720 | PA2398 | *fpvA* | ferripyoverdine receptor | 3.279750734 | 0.0142432 | 4.603218873 | 0.001216 |
| PA14_RS13700 | PA2403 |  | hypothetical protein | 2.712806524 | 0.0492968 | 5.340693847 | 9.1E-05 |
| PA14_RS13695 | PA2404 |  | hypothetical protein | 7.904163169 | 5.463E-08 | 14.84728002 | 1.18E-14 |
| PA14_RS13690 | PA2405 |  | hypothetical protein | 7.096982252 | 0.0007888 | 13.8417585 | 1.1E-08 |
| PA14_RS13685 | PA2406 |  | hypothetical protein | 5.775939288 | 0.0083335 | 11.91640815 | 8E-05 |
| PA14_RS13680 | PA2407 |  | adhesion protein | 7.048946537 | 1.662E-10 | 9.149766272 | 1.7E-14 |
| PA14_RS13675 | PA2408 |  | ABC transporter ATP-binding protein | 12.73065149 | 8.393E-08 | 16.33248958 | 2.85E-13 |
| PA14_RS13670 | PA2409 |  | ABC transporter permease | 6.258592181 | 7.84E-07 | 11.5385351 | 1.83E-10 |
| PA14_RS13665 | PA2410 |  | hypothetical protein | 5.313705124 | 3.162E-06 | 8.489965827 | 3.74E-11 |
| PA14_RS12095 | PA2664 | *fhp* | nitric oxide dioxygenase | 0.275000211 | 0.0034967 | 0.198170346 | 0.017984 |
| PA14_RS10940 | PA2864 |  | hypothetical protein | 0.494618165 | 0.00118 | 0.371369003 | 3.72E-07 |
| PA14_RS10390 | PA2971 |  | hypothetical protein | 2.758570401 | 3.452E-05 | 2.069504393 | 0.006 |
| PA14_RS09600 | PA3126 | *ibpA* | heat-shock protein IbpA | 0.314121003 | 3.095E-06 | 0.121572549 | 1.64E-17 |
| PA14_RS09065 | PA3231 |  | hypothetical protein | 5.877511917 | 0.0212953 | 0.479767295 | 0.002048 |
| PA14_RS07955 | PA3418 | *ldh* | leucine dehydrogenase | 0.450917344 | 0.0023841 | 0.349117886 | 2.23E-05 |
| PA14_RS07060 | PA3613 |  | hypothetical protein | 0.482527432 | 4.948E-06 | 0.457223336 | 5.85E-05 |
| PA14_RS05730 | PA3842 |  | chaperone | 6.434229731 | 1.114E-08 | 11.38431254 | 4.76E-15 |
| PA14_RS05300 | PA3919 |  | hypothetical protein | 0.494166532 | 0.0009442 | 0.462777522 | 0.004022 |
| PA14_RS04940 | PA3990 |  | hypothetical protein | 4.210839393 | 1.734E-05 | 9.163626103 | 2.32E-11 |
| PA14_RS04780 | PA4022 |  | aldehyde dehydrogenase | 0.248459575 | 4.475E-07 | 3.358944648 | 2.24E-07 |
| PA14_RS03805 | PA4218 |  | transporter | 0.110892568 | 2.086E-05 | 0.46833709 | 0.415038 |
| PA14_RS03790 | PA4221 | *fptA* | Fe(III)-pyochelin outer membrane receptor | 0.148961945 | 0.0212809 | 0.407121752 | 0.472711 |
| PA14_RS03785 | PA4222 |  | ABC transporter ATP-binding protein | 0.089478947 | 0.0001555 | 0.417648386 | 0.385847 |
| PA14_RS03780 | PA4223 |  | ABC transporter ATP-binding protein | 0.148795322 | 0.0046014 | 0.49330349 | 0.538955 |
| PA14_RS03770 | PA4225 | *pchF* | pyochelin synthetase | 0.137009602 | 0.0134075 | 0.459986784 | 0.56383 |
| PA14_RS03755 | PA4228 | *pchD* | pyochelin biosynthesis protein PchD | 0.147682949 | 0.0710893 | 0.441950285 | 0.625492 |
| PA14_RS03750 | PA4229 | *pchC* | pyochelin biosynthetic protein PchC | 0.211314429 | 0.0938911 | 0.36927466 | 0.447831 |
| PA14_RS03745 | PA4230 | *pchB* | isochorismate-pyruvate lyase | 0.127348738 | 0.029701 | 0.381685721 | 0.504952 |
| PA14_RS03715 | PA4236 | *katA* | catalase | 0.176236843 | 0.1218023 | 0.09315204 | 0.025992 |
| PA14_RS23140 | PA4366 | *sodB* | superoxide dismutase | 0.413700282 | 0.0327984 | 0.445349138 | 0.038681 |
| PA14_RS23245 | PA4386 | *groES* | co-chaperonin GroES | 0.270210221 | 0.0004152 | 0.352777827 | 0.004661 |
| PA14_RS23250 | PA4387 | *fxsA* | FxsA protein | 0.47622804 | 0.0179585 | 0.252634588 | 9.38E-06 |
| PA14_RS23690 | PA4475 |  | hypothetical protein | 0.476529884 | 0.0612141 | 0.314235912 | 0.002309 |
| PA14_RS24720 | PA4573 |  | hypothetical protein | 2.240826758 | 0.0101423 | 0.366681245 | 2.49E-08 |
| PA14_RS24795 | PA4587 | *ccpR* | cytochrome c551 peroxidase | 0.210215069 | 1.469E-10 | 0.227868785 | 1.05E-07 |
| PA14_RS24920 | PA4611 |  | hypothetical protein | 0.323971714 | 8.689E-06 | 0.420413163 | 0.001717 |
| PA14_RS25265 | PA4674 |  | virulence-associated protein | 7.693290922 | 5.277E-32 | 15.5115806 | 8.12E-63 |
| PA14_RS25745 | PA4761 | *dnaK* | molecular chaperone DnaK | 0.320795808 | 0.0131229 | 0.30818866 | 0.00329 |
| PA14_RS25750 | PA4762 | *grpE* | heat shock protein GrpE | 0.373669252 | 0.1339316 | 0.268516207 | 0.020214 |
| PA14_RS27250 | PA5053 | *hslV* | ATP-dependent protease peptidase subunit | 0.342991972 | 0.0142955 | 0.239790511 | 0.000252 |
| PA14_RS27255 | PA5054 | *hslU* | ATP-dependent protease ATP-binding subunit HslU | 0.283070086 | 0.0001054 | 0.326577717 | 9.48E-05 |
| PA14_RS27855 | PA5171 | *arcA* | arginine deiminase | 0.169963127 | 7.549E-05 | 0.215255278 | 0.002734 |
| PA14_RS27860 | PA5172 | *arcB* | ornithine carbamoyltransferase | 0.108602065 | 3.72E-06 | 0.196245682 | 0.003216 |
| PA14_RS27865 | PA5173 | *arcC* | carbamate kinase | 0.145259796 | 2.995E-06 | 0.18992584 | 0.000397 |
| PA14_RS28585 | PA5314 |  | hypothetical protein | 2.331506519 | 0.0001657 | 4.019648173 | 1.66E-11 |
| PA14_RS28785 | PA5355 | *glcD* | glycolate oxidase subunit GlcD | 0.429714848 | 2.085E-07 | 0.362355035 | 1.84E-07 |
| PA14_RS29170 | PA5427 | *adhA* | alcohol dehydrogenase | 0.416262587 | 1.401E-05 | 0.193252602 | 7.61E-14 |
| PA14_RS29420 | PA5475 |  | hypothetical protein | 0.456836358 | 3.745E-07 | 0.336759075 | 1.74E-11 |
| PA14_RS29775 | PA5546 |  | hypothetical protein | 0.426662698 | 9.17E-05 | 0.358275557 | 2.47E-06 |
| PA14_RS03200 |  |  | holin | 0.419918976 | 0.0142306 | 0.433628797 | 0.001492 |
| PA14_RS03800 |  |  | hypothetical protein | 0.196962633 | 0.00366 | 0.446508808 | 0.388061 |
| PA14_RS05135 |  |  | hypothetical protein | 0.31548681 | 4.431E-07 | 0.263519546 | 1.85E-09 |
| PA14_RS05725 |  |  | hypothetical protein | 6.426203561 | 6.248E-14 | 2.921462195 | 1.68E-06 |
| PA14_RS08970 |  |  | hypothetical protein | 2.149475732 | 0.4760699 | 0.428333269 | 0.031764 |
| PA14_RS08990 |  |  | hypothetical protein | 2.730234982 | 1.153E-08 | 8.775835031 | 1.26E-35 |
| PA14_RS10080 |  |  | tRNA-Pro | 0.352325009 | 0.9622377 | 0.271609018 | 0.051529 |
| PA14_RS11000 |  |  | tRNA-Ser | 2.653059021 | 0.7699042 | 0.465473958 | 0.836408 |
| PA14_RS12750 |  |  | hypothetical protein | 0.33012309 | 6.865E-05 | 0.28191416 | 1.25E-07 |
| PA14_RS14975 |  |  | hypothetical protein | 6.489454385 | 0.8590528 | 0.487879538 | 0.03177 |
| PA14_RS16085 |  |  | hypothetical protein | 0.307659363 | 3.745E-07 | 0.103214549 | 2.94E-17 |
| PA14_RS17290 |  |  | type III secretion system needle lengthdeterminant | 5.597428871 | 9.138E-16 | 4.601647944 | 1.61E-14 |
| PA14_RS18020 |  |  | hypothetical protein | 0.316061252 | 0.0003728 | 0.285355337 | 0.000327 |
| PA14_RS20955 |  | *spcU* | SpcU | 3.09291298 | 9.69E-10 | 4.959978228 | 1.31E-20 |
| PA14_RS20960 |  | *exoU* | ExoU | 4.443031844 | 1.565E-21 | 8.603025268 | 1.97E-44 |
| PA14_RS21810 |  |  | hypothetical protein | 0.234833577 | 4.207E-07 | 0.259945361 | 8.21E-06 |
| PA14_RS24765 |  |  | tRNA-Arg | 4.883418724 | 0.0036956 | 4.762701306 | 0.003424 |

E: exponential growth phase

S: stationary growth phase
