# Supplementary material for: HigB of Pseudomonas aeruginosa Enhances Killing of Phagocytes by Up-Regulating the Type III Secretion System in Ciprofloxacin Induced Persister Cells
Source: Front Cell Infect Microbiol. 2016 Oct 14;6:125. doi: 10.3389/fcimb.2016.00125 (PMC5064212; doi:10.3389/fcimb.2016.00125)
Supplement: Table S2 — Bacterial strains, plasmids and primers used in this study. [file Table2.DOCX]

Table S2. Bacterial strains, plasmids and primers used in this study.

| **Strain/ Plasmid /Primer** | **Description** | | **Source (Reference)** |
| --- | --- | --- | --- |
| ***P. aeruginosa*** | | | |
| PA14 | | Wild type strain of *Pseudomonas aeruginosa* | ([Liberati et al., 2006](#_ENREF_3)) |
| *higA*::Tn | | PA14 with MAR2xT7 transposon inserted at *higA*; Gm^R^ | ([Liberati et al., 2006](#_ENREF_3)) |
| *exsA*::Tn | | PA14 with MAR2xT7 transposon inserted at *exsA*; Gm^R^ | ([Liberati et al., 2006](#_ENREF_3)) |
| *higA*::Tn /Tn7T-*higA* | | *higA*::Tn with *higA* inserted on chromosome with mini-Tn7T insertion; Tc^r^ | This study |
| △*higB* | | PA14 deleted of *higB* | This study |
| △*higB*△*higA* | | PA14 deleted of *higB* and *higA* | This study |
| **Plasmid** | |  |  |
| pUCP20 | | *Escherichia*–*Pseudomonas* shuttle vector without *lac* promoter; Ap^r^ | ([Choi and Schweizer, 2006](#_ENREF_1)) |
| pEX18Tc | | Gene replacement vector; Tc^r^, *oriT*^+^, *sacB*^+^ | ([Choi and Schweizer, 2006](#_ENREF_1)) |
| pMMB67EH | | Expression vector with *tac* promoter; Ap^r^ | ([Fürste et al., 1986](#_ENREF_2)) |
| pUC18T-mini-Tn7T-Tc | | mini-Tn7 base vector from insertion into chromosome attTn7 site; Tc^r^ | ([Choi and Schweizer, 2006](#_ENREF_1)) |
| pUC18T-mini-Tn7T-Tc-*higA* | | pUC18T-mini-Tn7T-Tc with *higA*; Tc^r^ | This study |
| pEX18Tc-△*higB* | | *higB* gene of PAK deletion on pEX18Tc; Tc^r^ | This study |
| pEX18Tc-△*higB*△*higA* | | *higB* and *higA* gene of PAK deletion on pEX18Tc; Tc^r^ | This study |
| pMMB67EH-*higA* -His | | *higA* gene with His-tag driven by *tac* promoter on pMMB67EH; Ap^r^ | This study |
| pMMB67EH-*higB* -His | | *higB* gene with His-tag driven by *tac* promoter on pMMB67EH; Ap^r^ | This study |
| pUCP20-P*_higB_*::*gfp* | | *higA* promoter of PA14 fused to *gfp* on promoterless pUCP20; Ap^r^ | This study |
| pUCP20-P*_higB_*::*mcherry* | | *higA* promoter of PA14 fused to *mcherry* on promoterless pUCP20; Ap^r^ | This study |
| pUCP20-P*_exoU_*-*exoU*-His | | *exoU* promoter of PA14 fused to *exoU-His* on promoterless pUCP20; Ap^r^ | This study |
| pRKaraRed-P*_exoU_*-*exoU*-His | | *exoU* promoter of PA14 fused to *exoU-His* on promoterless pRKaraRed; Tc^r^ | This study |
|  | |  |  |
| **Primer** | | **Sequence (5’→3’)** | **Function** |
| KpnⅠ-higB-F | | CGGGGTACCAGTGAAGTTAACGCTTAACGTTAAG | *higB* cloning |
| HindⅢ-higB-R | | CCCAAGCTTTCAGTGGTGGTGGTGGTGGTGACCTCCGTGGTAATCAACTATTTCGACTTC | *higB* cloning |
| BamHⅠ-higA-F | | CGCGGATCCAAATAGTTGATTACCACTGAGGAGGTGG | *higA* cloning |
| HindⅢ-higA-R | | CCCAAGCTTCTAGTGGTGGTGGTGGTGGTGACCTCCTCCGTGAGCAAGCAGCGG | *higA* cloning |
| SacⅠ-higBhigAup-F | | TCGATGGAGCTCTAGCGGATGGTGGGGAAGGG | *higB* and *higA* deletion |
| KpnⅠ-higBhigAup-R | | CGGGGTACCATGCCCCGCTCCATCCCTTC | *higB* and *higA* deletion |
| KpnⅠ-higBhigAdown-F | | CGGGGTACCCGGTGACGTTGATCGTAGAGCCC | *higB* and *higA* deletion |
| HindⅢ-higBhigAdown-R | | CCCAAGCTT CATCCCCCACTTCACCGAGGG | *higB* and *higA* deletion |
| KpnⅠ-higBdown-F | | CGGGGTACCATGGCTACCAATGGTATGCGCCC | *higB* deletion |
| HindⅢ-higBdown-R | | CCCAAGCTT CCGTGTGATCCGTGCCGGC | *higB* deletion |
| KpnⅠ-exoU-F | | CGGGGTACCGCTGGACGAGATGGCGCG | *exoU* cloning |
| HindⅢ-exoU-R | | CCCAAGCTTTCAGTGGTGGTGGTGGTGGTGACCTCCTGTGAACTCCTTATTCCGCCAAGC | *exoU* cloning |
| EMSA-(M)-F | | CTGTAGTGAAGTCTAGACTTAAGCATATGGGTTAATGA | EMSA |
| EMSA-(M)-R | | TCATTAACCCATATGCTTAAGTCTAGACTTCACTACAG | EMSA |
| EMSA-F | | CTGTAGTGAAGTTAACGCTTAACGTTAAGGGTTAATGA | EMSA |
| EMSA-R | | TCATTAACCCTTAACGTTAAGCGTTAACTTCACTACAG | EMSA |
| HindⅢ-gfp-F | | CCCAAGCTTATGAGTAAAGGAGAAGAACTTTTCACTG | *gfp* cloning |
| PstⅠgfp-R | | AACTGCAGTTATTTGTATAGTTCATCCATGCCATG | *gfp* cloning |
| HindⅢ-mcherry-F | | CCCAAGCTTATGGTCAGCAAGGGAGAGGAAG | *mcherry* cloning |
| BamHⅠ-mcherry-R | | CGCGGATCCCTATTTGTATAATTCGTCCATTCCACC | *mcherry* cloning |
| KpnⅠ-Pro-higB-F | | CGGGGTACCGAGGAGATTTCCGAGTTGCTGTTG | *higB* promoter cloning |
| HindⅢ-Pro-higB-R | | CCCAAGCTTTAACCCTTAACGTTAAGCGTTAACTTC | *higB* promoter cloning |
| 5-AP | | CGACTTGAACTCCAGGGGGGGGGGG | 5’Race |
| 5-NP | | CGACTTGAACTCCAGG | 5’Race |
| higB-R-1 | | TCAACTATTTCGACTTCTT | 5’Race |
| higA-R-1 | | GTACTCACTCTGGAGATTCATC | 5’Race |
| higB-F | | TTTGAGACGGGTCTTTCG | RT-PCR |
| higB-R | | TAGCCGCATGAAGCATTG | RT-PCR |
| higA-F | | GTTTCTGATGGAGTTGGAT | RT-PCR |
| higA-R | | ATATCGTTCACTGTCGGA | RT-PCR |
| PA1769-F | | GAACATCAGCTTCGTCAA | RT-PCR |
| PA1769-R | | TCGGCAGCATTATTGATT | RT-PCR |
| exsA-F | | GCTATGTCGTAAGTACCA | RT-PCR |
| exsA-R | | GAAGCCTTGTAGAAACTG | RT-PCR |
| exsC-F | | ATGGATTTAACGAGCAAGGTCAA | RT-PCR |
| exsC-R | | GAGGGACAGGGAAGGCAAA | RT-PCR |
| exoU-F | | GTTGAGTGCTTACATTCC | RT-PCR |
| exoU-R | | TTGAACACCACTAATTGC | RT-PCR |
| pcrV-F | | CACGCTCTATGGCTATGC | RT-PCR |
| pcrV-R | | AAGGTATCCAGATTGCTCAG | RT-PCR |
| PA0668.1-F | | AAGGTCTTCGGATTGTAA | RT-PCR |
| PA0668.1-R | | GTGCTTATTCTGTTGGTAA | RT-PCR |

**References**

Choi, K.-H., and Schweizer, H.P. (2006). mini-Tn7 insertion in bacteria with single attTn7 sites: example Pseudomonas aeruginosa. *Nature protocols,* 1**,** 153-161.

Fürste, J.P., Pansegrau, W., Frank, R., Blöcker, H., Scholz, P., Bagdasarian, M., and Lanka, E. (1986). Molecular cloning of the plasmid RP4 primase region in a multi-host-range tacP expression vector. *Gene,* 48**,** 119-131.

Liberati, N.T., Urbach, J.M., Miyata, S., Lee, D.G., Drenkard, E., Wu, G., Villanueva, J., Wei, T., and Ausubel, F.M. (2006). An ordered, nonredundant library of Pseudomonas aeruginosa strain PA14 transposon insertion mutants. *Proc Natl Acad Sci U S A,* 103**,** 2833-2838.
